# Detection of Mechanically Separated Meat from Pork in Meat-Containing Foods by Targeted LC-MS/MS Analysis

**DOI:** 10.3390/foods14081317

**Published:** 2025-04-10

**Authors:** Christian Wilhelm, Mikko Hofsommer, Nadine Fischbach, Stefan Wittke

**Affiliations:** 1 Department 1 Technology, University of Applied Sciences Bremerhaven, An der Karlstadt 8, 27568 Bremerhaven, Germany; cwilhelm@hs-bremerhaven.de; 2 Gesellschaft für Lebensmittel-Forschung mbH, Landgrafenstraße 16, 10787 Berlin, Germany; hofsommer@gfl-berlin.com (M.H.); fischbach@gfl-berlin.com (N.F.)

**Keywords:** mechanical separated meat (MSM), pork, LC-MS/MS, food fraud, food authenticity

## Abstract

Microscopy and calcium analysis have proven to be inadequate for the detection of mechanically separated meat (MSM) in meat-containing foods. Therefore, a pseudo-MRM-LC-MS/MS-based bottom-up assay was developed and validated for the detection of porcine MSM. In contrast to a comparable study on MSM from poultry, the studies on porcine MSM showed that the use of cartilage/intervertebral disc material was not useful. Here, we report a new marker protein from porcine MSM, protegrin-4, which allows the detection of 5/3/1 mm MSM. The validity of the developed assay was ensured by the investigation of 182 blinded samples. After unblinding, all samples containing 5/3/1 mm MSM and all negative control samples were correctly classified (precision 100%). Furthermore, new results related to the investigation of the species specification of chicken, turkey, and pork are presented. In conclusion, LC-MS/MS-based detection of potentially undeclared use of MSM has been successfully extended from poultry to porcine MSM. Moreover, the assay was successfully transferred to a tripleQuad LC-MS system.

## 1. Introduction

In the food industry, the replacement of high-quality raw materials with cheaper substitute products can be observed time and again [1]. According to EU Regulation 853/2004, MSM is mechanically separated from the bone with a destroyed, dissolved or altered muscle fiber structure [2]. In 2011 alone, 64,000 tons of MSM from poultry and pork were produced in Germany, respectively [3]. However, only half of the MSM produced was exported to third countries. The EU Commission therefore states that the whereabouts of a total of around 65,000 tons of MSM from poultry and pigs, respectively, are unknown each year (plus an unknown amount of MSM from poultry wishbone) [3]. This is particularly problematic because there is a lack of adequate analytical methods that can specifically detect the undeclared use of MSM in foods.

Depending on the food to be analyzed, the demands on the techniques used can be very high due to the complex matrix, the origin from different sources, the composition (proteins, lipids, carbohydrates, salts, etc.), or the degree of processing. With regard to MSM, it is known that the official testing methods [4] are unsuitable ([5], personal communication). For instance, only increased bone content due to the extraction technology would allow identification of MSM via calcium determination or microscopy [6,7,8]. However, calcium contents can be adjusted by technical methods to inconspicuous values. In addition, although some standard histological methods, such as silver nitrate staining of not-decalcified bone tissue (Van Kossa method, ASU 06.00-13:1989-12) and “Alcian Green”-staining of cartilage tissue (ASU 06.00-13:1989-12), are available, both methods do not always lead to unambiguous results. This is particularly the case if the MSM is “baadered” after the actual production or if bone fragments, cartilage, and connective tissues are removed using sieves with 3 mm perforations, e.g., after the extraction of wishbone meat from poultry ([9], personal communication). Hence the question arises to what extent such problems can be avoided with new methods in food monitoring and which platforms are suitable. For this purpose, it must be ensured that food monitoring can use the newly developed methods at all (technical requirements) or is allowed to do so (regulatory requirements). The latter is anchored in Regulation (EU) 2017/625, according to which the methods should ideally be official test methods that have been validated (in interlaboratory tests) in accordance with the specifications in Annex III of the aforementioned regulation (Chapter IV, Section 34 (2a)). However, if adequate technologies are missing, even new developed methods can be implemented (Chapter IV, Section 2b) [10].

The advantage of mass spectrometry over other methods are certainly the precise, reliable and specific results. In particular, tandem mass spectrometry, especially in combination with established chromatographic techniques (LC-MS/MS), can be a useful tool for food authentication [11,12,13,14,15,16,17,18].

Tian et al. developed an LC-QTOF-MS-based approach for the botanical discrimination of honeys [19], and Beteinakis et al. performed similar research for the authentication of table olives, combining LC-HRMS and NMR [20]. In this context, Hu et al. described the differentiation of three commercial tuna species through GC-Q-TOF and UPLC-Q/Orbitrap mass spectrometry-based metabolomics and chemometrics. A total of 22 out of 77 metabolites were identified, and chemometrics revealed that 38 metabolites might serve as potential biomarkers [21]. Other approaches, reviewed by Zhong et al. and Xu et al., allow fruit juice authenticity assessments by targeted and untargeted analyses such as LC-MS, GC-MS, etc. [22,23]. Furthermore, Stader et al. used LC-MS/MS for the detection of the undeclared addition of plasma proteins (especially from pigs) in sausage products [17].

Additionally, Wilhelm et al. presented an LC-MS/MS-based method that allows the detection of poultry MSM in sausage and meat products based on cartilage collagen (COL2A1) [24]. This method was recently supported by a ruling of the German Federal Administrative Court (Ref.: 3C 4.22), determining that the common usage of processed wishbone meat in food industry should be declared as the use of MSM in accordance with EU Regulation 853/2004 [2]. According to the poultry industry, this new declaration would lead to double-digit million losses, as consumers would refuse to eat food containing mechanically separated meat ([9], personal communication]. Similar statements were made by the British Poultry Council, according to which wishbone meat would become “effectively valueless”, as there would be no market for it in the UK, and therefore, up to 10,000 tons of meat would have to be thrown away, resulting in total costs of over 75 million pounds [25].

In contrast to poultry, there are currently no adequate methods available for the detection of porcine MSM. Although R.A. Field describes various methods for the qualitative and quantitative determination of bone marrow in mechanically separated meat [26], these methods are, to our knowledge, not applied in food control. On the other hand, the histological methods and the detection of calcium require little time and technical effort [27] but are unsuitable, as these methods are known to not provide reliable results [28].

In conclusion, the same challenges have to be solved for the detection of porcine MSM as for poultry MSM [24]. In this case—similar to poultry MSM—intervertebral disc and cartilage components could be of interest, as they cannot be completely separated and should inevitably occur even in MSM produced with low pressure or “baadered” MSM. In pigs, the proteome of intervertebral discs and cartilage also consists predominantly of collagens and can therefore be easily distinguished from other tissue types [27].

In addition, in pigs, as in all vertebrates that have bones with medullary cavities (e.g., long bones) or spongy bone structures (spongiosa), proteins released during the breakdown of the bone structure may be of interest [28]. Lipid, cholesterol, nucleic acids, hemoglobin, and pH values increase with bone marrow, whereas the myosin and actin (muscle proteins) content decreases. According to Fields [26], these are insufficiently specific since the actual amount of marrow in meat from advanced meat recovery systems is not known.

Another opportunity could be proteins or peptides from leukocytes, e.g., protegrins, as antimicrobial peptides [29,30]. In vertebrates, leukocytes are found not only in blood and the lymphatic organs but also in bone marrow [31]. As the lymphatic organs and blood (except for blood sausages or in the case of undeclared addition) are not usually used for the production of sausage products, the remaining source would be bone marrow. This could only be accessed by destroying the bone structure and therefore might serve as an indicator for the use of mechanically separated meat.

**Figure 1:**
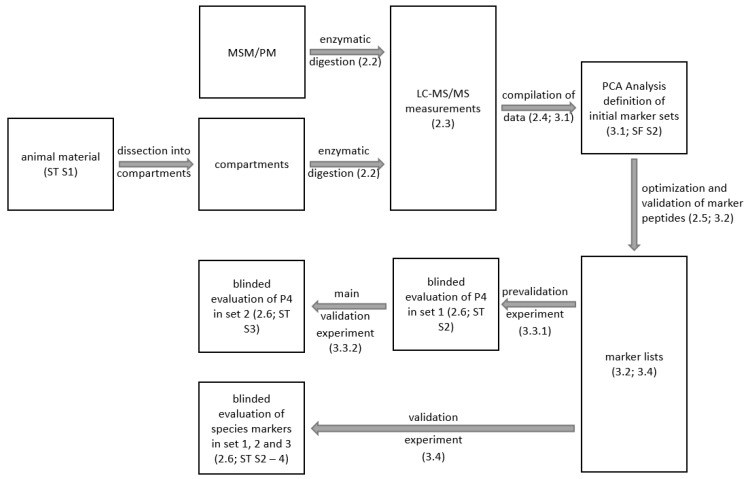
Graphical scheme of the developed assay. In the first step, the animal material samples (Supplementary Figure S1) were dissected into compartments of different tissue types, and the proteins/peptides in the individual compartments as well as in processed meat (PM) and MSM were detected by LC-MS (2.3) after enzymatic digestion (2.2). A database (MarkerView) was used to summarize (cluster) the measurements according to their type/compartments (2.4; 3.1). Subsequently, the detected ions were compared with each other, resulting in a group of 50 potential marker peptides that could distinguish between MSM and PM and all other compartments with high precision, shown by PCA (3.1; Supplementary Figure S2). Subsequently, the three best marker ions were defined based on the selection rules (2.4), of which only one marker peptide was used after the final optimization steps (2.5; 3.2). The specificity of the marker peptide was then validated by examining 26 samples (set 1; Supplementary Table S2) with defined MSM contents (1, 3, 5, and 8 mm; 0, 10, and 20%) in a blinded study and then by examining 51 samples from retail and butcher’s sausages (set 2; Supplementary Table S3) in a second blinded validation study. The assay for species determination was developed analogously with a final blinded validation study by examining 182 samples (sets 1, 2, and 3; Supplementary Tables S2–S4).

In summary, the aim of this study is to develop a method based on marker proteins/peptides using LC-MS/MS to specifically detect the use of porcine MSM in sausage and meat products. The methodology of the LC-MS/MS method for the detection of MSM in pigs (Figure 1) is to a large extent analogous to the method used by Wilhelm et al. [24]. Again, this includes the untargeted examination of different tissue types (skin, muscle, tendon, cartilage, etc.), porcine MSM of different quality (1–8 mm MSM), and the targeted blinded validation of the final subset of marker peptides.

The primary hypothesis is that porcine MSM can be detected using cartilage-specific proteins (e.g., type II collagens), as in poultry [24]. However, alternative proteins were also be investigated to define the most appropriate detection method. The advantage of this (un)targeted approach is that additional information on species authenticity (chicken, turkey, and pork) can be obtained by examining the different tissue types. The results are also presented against the background of questions from different religious communities as to the extent to which a food is halal [32] or kosher.

## 2. Materials and Methods

### 2.1. Animal and Raw Material

Fifteen pig tails and four pig backbones from different animals and different breeds (“Duroc”, “Landschwein” and “Edelschwein”) were used, from which pure tissue-type material (skin, tendons, cartilage/intervertebral discs and muscle meat; Supplementary Figure S1) was obtained. In addition, pure skin and tendon material from 10 other pigs (“Duroc” and “Edelschwein”) as well as four different types of MSM (1, 3, 5, and 8 mm sieve hole diameter) and industrially processed pork meat were also used for this study.

All animal material samples from pigs were obtained from regional butchers, the MSM from pigs was obtained from Hovu B.V. (NL 664 EG, Van Leeuwenhoekweg 38, 5482 Schijndel, The Netherlands), and the processed meat was purchased wholesale.

Furthermore, 35 whole chickens, 10 fresh chicken thighs, 12 turkey legs (including eight lower legs and two upper legs), and 4 turkey carcasses were used to obtain pure tissue material (breast/thigh meat, skin, tendons and cartilage/vertebral disc) (Supplementary Table S1). All chicken samples and the 12 turkey legs were purchased from retailers in and around Bremerhaven (Germany), while the 4 turkey carcasses were obtained from Bartels GmbH & Co. KG (Langenberger Str. 125, 33,397 Mastholte, Germany).

After dissection, the sample material was freeze-dried (24 h, Alpha 1-2 LDplus, Christ GmbH, Osterode am Harz, Germany), ground in a mixer (Nutrition Mixer, DS Produkte GmbH, Stapelfeld, Germany), and stored at −20 °C until further use.

In order to exclude further sources of entry of potential markers for MSM, especially from blood components that find their way into the product through the use of bone marrow, three dried blood plasmas (DBPs) from different manufacturers were also examined. This included samples from Acontex GmbH (In der Mark 2, 33,378 Rheda-Wiedenbrück, Germany), Foodchem International Corporation (2277 Zuchongzhi Road, 201,203 Shanghai, China), and Xiamen Hyfine Gelatine Co., Ltd. (14 Mid-Liushan Road, 361,000 Haicang, China).

### 2.2. Sample Preparation

The enzymatic digestion with thermolysin (Figure 1) was carried out for all samples, except the DBPs in this study, according to Wilhelm et al. [24] with slight adaptions. In brief, 5 g of the lyophilized sample was mixed with 20 mL ultra-pure water (PURELAB™ flex 2; ELGA LabWater, Veolia Water Technologies Deutschland GmbH, Celle, Germany). After heat-denaturation (95 °C, 10 min) the sample was homogenized (Ultra-Turrax, 15,000 rpm, 1 min), and an aliquot of 2 mL (equivalent to 0.5 g lyophilized sample) was transferred to 6 mL ultra-pure water. Subsequently, 3 mg thermolysin (CAS-Nr: 9073-78-3, Geobacillus stearothermophilus; Sigma-Aldrich, Hamburg, Germany) in 2 mL ultra-pure water was added. After incubation (4 h, 37 °C), the enzyme was deactivated by heat denaturation (95 °C, 20 min), centrifugated (3800× *g*, 10 min, Universal 320R; Hettich GmbH, Tuttlingen, Germany), and filtered (Rotilabo^®^-syringe filters, 0.20 µm, mixed cellulose ester (CME), Carl Roth GmbH+Co.KG, Karlsruhe, Germany). Finally, the protein content of the supernatant was adjusted to 2 mg/mL [15]. The samples were measured directly by LC-MS/MS or stored at 4 °C (maximum 7 days) until further use.

The DBPs were also processed according to Wilhelm et al. [24] with few adaptions. After determining the protein content using the BCA-assay (Pierce^TM^ BCA Protein Assay Kit, Fisher Scientific GmbH, Schwerte, Germany), an amount equivalent to 0.3 g of total protein was mixed with 8 mL ultra-pure water by vortexing (Vortex-Mixer, 2000 rpm, 30 s) and shaking (Rotator Genie, speed 4, 1 h). The sample was then heat-denatured (95 °C, 10 min) and digested with thermolysin as described above.

### 2.3. LC-MS/MS

LC-MS and LC-MS/MS (Figure 1) were carried out as described by Wilhelm et al. [24]. In brief, a M5 microLC-system online coupled to a TripleTOF 4600 (AB Sciex, Darmstadt, Germany) was used, equipped with a HALO Fused-Core C18 LC column (35 °C, 50 × 0.5 mm, 2.7 µm, 90 A; MZ Analysentechnik, Mainz, Germany). An aliquot of 5 µL of sample was injected for each analysis utilizing the LC gradient described by Wilhelm et al. [24].

Peptides were analyzed in the Information Dependent Acquisition (IDA) or pseudo-Multiple Reaction Monitoring (pMRM; Supplementary Table S5) workflow using the Analyst software (v1.7.1; AB Sciex, Darmstadt, Germany). Nitrogen was used as the collision gas for collision-induced fragmentation (CID).

The pMRM transitions were reconstructed by the software [33,34,35] using the full-scan fragment ion spectra of each precursor.

### 2.4. Definition of Specific Marker Ions (MarkerView)

The compilation of the individual datasets according to the tissue type was performed using MarkerView (V.1.2.1; AB Sciex, Darmstadt, Germany, Figure 1). The resulting peak list characterizes the detected ions by their m/z-ratio, molecular weight (kDa), and signal intensity. Ions from different samples were regarded as identical if the m/z-deviation was equal to or less than ±0.005 Da and if the deviation of the retention time was equal to or less than ±0.1 min.

The tissue type-specific marker ions were then defined by comparing the individual sample (tissue) types against all other samples (*p* < 0.05; *t*-test). In addition, the following quality-control criteria were adapted:

1. One-fold-charged molecules are not accepted as candidates;
2. The frequency of occurrence of every candidate marker ion must be 100% in one of the groups (meat, tendon, skin, MSM, etc.);
3. Four pMRM transitions could be allocated;
4. In pure tissue, each pMRM transition must be at least ten times higher than the minimum acceptable signal intensity of 50 counts or SNR of 3 (Supplementary Table S7);
5. Marker ions identified as peptides must show a minimal size of six amino acids.

The marker ion patterns were used to distinguish the different subgroups (skin, meat, processed meat, cartilage, and MSM) by principal component analysis (Figure 1, PCA, Supplementary Table S6) [15,24,35].

### 2.5. Identification and Verification of Specific Marker Ions

ProteinPilot (V 5.0, AB Sciex, Darmstadt, Germany) using the latest UniProt database (7 October 2024) allowed automated protein identification (Paragon search engine [36]). Manual annotation (Δ m/z = ±0.005 Da) of MS/MS spectra was performed using the Bio Tool Kit V. 2.2.0 (AB Sciex, Darmstadt, Germany).

All sequences were examined for correct species assignment using Protein Blast (https://blast.ncbi.nlm.nih.gov/Blast.cgi, last accessed on 6 March 2025).

### 2.6. Samples for the Validation of the MSM Assay and the Species-Specific Markers

The validity of the MSM assay was tested by a total of 77 samples with unknown composition divided into two separated sets (set 1/2, n = 26/51) in a blinded fashion.

The first set (Supplementary Table S2) of 18 negative control (NC1) and 8 positive control (PC1) samples containing 10 and 20% of 1, 3, 5, and 8 mm MSM, respectively, was used to pre-validate the markers for porcine MSM.

The final validation for the MSM assay was performed with a second set (Supplementary Table S3) of 43 negative control samples (NC2) and 8 positive control samples (PC2), 5 of which had an unknown declared MSM content, and the other 3 samples had MSM contents of 7.5, 15, and 20%.

The samples were partially produced by various butchers (n = 59 in northern Germany) or acquired as industrially produced and commercially available sausages from different supermarkets (n = 18).

A third set of 105 samples (Supplementary Table S4) not containing MSM was used together with sets 1 and 2 (182 samples in total) to validate the species-specific markers. These samples were also partly produced by regional butchers (n = 52) and partly from industrial sources (n = 53) and contained different compositions and proportions of chicken, turkey, and pork or were produced exclusively with meat of a single species. This procedure ensures the analytical validity of the sample material, which is required by EU Regulation No. 2017/625 [14].

All samples from the three sets were investigated in duplicates according to the standardized sample preparation (lyophilization, homogenization and digestion) before LC-MS/MS analysis (repeatability).

### 2.7. Assignment of Samples

The MasterView^TM^ software tool (v.1.1, AB Sciex, Darmstadt, Germany) was used for the classification of the (blinded) samples with regard to the use of MSM meat and allocation to species and tissue type, respectively. For a positive assignment of a marker ion, a small difference in retention time (Δ t < ±0.1 min) and a high mass accuracy for the pMRM transitions (Δ m/z < ±0.005 Da) were mandatory. Only pMRM transitions showing signal intensities of SNR ≥ 3:1 were used, and in addition, all pMRM transitions had to exceed a predetermined intensity of 50 counts to avoid false-positive results (Supplementary Table S7; measurement uncertainty).

After classification, the samples were de-blinded, and the specificity and sensitivity (precision) were determined for sets 1 and 2 (MSM assay), as well as for sets 1, 2, and 3 for the species assay.

### 2.8. Statistical Analysis

Median, mean value, and standard deviation were determined in Excel^®^ (MS Office 2019, Microsoft, Redmond, WA, USA). MarkerView (v 1.1; AB Sciex, Darmstadt, Germany) was used for *t*-test (*p*-value < 0.05) and principal component analysis [15,24,37].

### 2.9. Calcium Content

The calcium contents were determined according to DIN EN 16943:2017-07 [38] in line with national standards and was carried out using optical emission spectrometry with inductively coupled plasma (ICP-OES) after microwave pressure digestion.

## 3. Results

As shown by Lasch et al. [15] and Wilhelm et al. [24], bottom-up proteomics is sufficiently reproducible to generate tissue-typical markers for an investigated species, and that set of markers can be used to detect these tissue types in more complex samples.

### 3.1. Definition of Specific Marker Ions for Porcine MSM

A total of 82 samples of different porcine tissue types (skin, meat, intervertebral disc, and cartilage; Supplementary Figure S1) as well as a total of 42 samples of processed meat and 4 types of MSM were investigated by LC-MS/MS.

**Figure 2:**
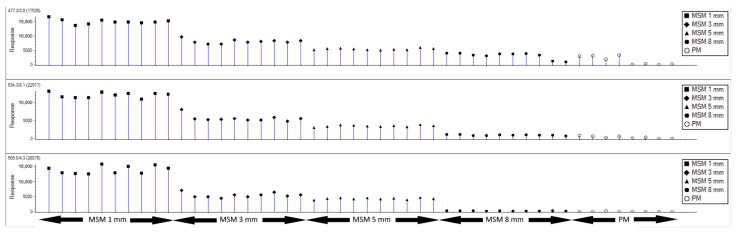
Response profiles of M1 (up), M2 (middle), and M3 (down) in MSM (1, 3, 5, and 8 mm) and processed meat (PM) samples after untargeted LC-MS analysis showing a gradual change with the highest values in MSM (8 mm) and the lowest values in processed meat (PM) for all investigated marker peptides.

These data were imported to MarkerView, clustered (m/z, RT), and sorted by tissue type. A total of 53,818 m/z-clusters were annotated, resulting in 6376 potential marker candidates with a *p*-value of <0.05. A subset of 50 potential markers (*p*-value < 7.548 × 10^−21^) was selected, resulting in the separation of the subgroup MSM from all other tissue types by principal component analysis (PCA, Supplementary Figure S2). Three of these potential marker ions (M1–M3) showed the highest discriminatory value, and Figure 2 compares their intensity profiles in MSM of different quality (1, 3, 5, and 8 mm; n = 10) and industrially processed meat (n = 8).

All three marker ions showed similar behavior depending on the different types of MSM and processed meat. Nonetheless, there were distinct differences between the three marker ions. M3 showed a regular decrease in intensity depending on the quality of the MSM, with the highest intensity in 1 mm MSM (mean of precursor: 13,914 ± 1259 cps), whereas M3 was barely detectable in 8 mm MSM (mean of precursor: 307 ± 61 cps) and was nearly undetectable in processed meat (SN < 3:1). Although M1 and M2 also showed this trend, it was much less pronounced in M2 between 3, 5, and 8 mm MSM, and both markers also showed higher intensity values in some cases in processed meat (M1 mean of precursor: 509 ± 282 cps; M2 mean of precursor: 268 ± 97 cps). It is important to note that the M3 content in processed meat (PM) was very low. Hence, processed meat could not provide a false-positive declaration. Furthermore, it needs to be emphasized that, contrary to the initial assumption that collagens could also be used as markers for porcine MSM, no collagens (fragments) were found to be discrepant in the definition of the potential biomarkers. The definition of respective marker candidates failed.

### 3.2. Identification of the Potential Marker Ions by LC-MS/MS

The MS/MS spectra of the marker ions M1 and M2 could be assigned to any known protein sequence neither with ProteinPilot nor by manual annotation (Table 1). This could, for example, be due to post-translational modifications (glycosylation, phosphorylation, disulfide bonds, etc.), which make identification more difficult.

For marker ion M3, a UniProt database match to the pro-peptide protegrin-4 (PG4_PIG) was received (Supplementary Figure S3). The sequence in question was checked by Protein Blast, showing only one further database hit for warthog (*Phacochoerus africanus*). In addition, the structural homology to the other known protegrins in pig (PG-1 to PG-5) was confirmed.

**Figure 3:**
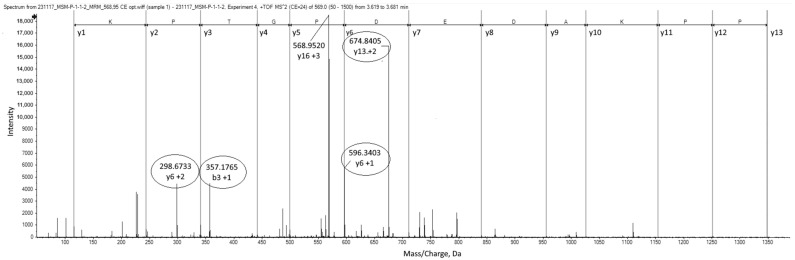
MS/MS spectrum for the marker peptide M3 (LDQPPKADEDPGTPKP; m/z 568.953; 3-fold charged; UniProt: PG4_PIG) in MSM (1 mm) after thermolysin digestion with optimized CE (24 V) showing the pMRM transitions (highlighted by circles) for identification and the sequence (y-series).

M3 was therefore defined as the most interesting marker peptide, and the measurement conditions were optimized for a subsequent targeted application (pMRM). The collision energy was optimized, and four characteristic fragment ions were defined for M3 (Figure 3, Table 1). These fragment ions highlighted by circles in Figure 3 were used in all further investigations.

### 3.3. Validation of M3

The validation of an analytical method should be characterized by features such as accuracy (trueness and precision), fitness for purpose (matrix and concentration range), limit of detection, limit of quantification, precision (ideally determined in interlaboratory comparisons according to ISO 5725), repeatability, reproducibility, recovery, selectivity, sensitivity, linearity, measurement uncertainty and other criteria selected as required [10].

Validation parameters such as linearity, recovery, and limit of quantification were not addressed because the method does not detect absolute levels. The quantification of M3 in different sample types from industrial production and from butchers is currently under development, but the transfer of the method to a second laboratory (tripleQuad 5500; AB Sciex, Darmstadt, Germany; reproducibility) was already successful.

#### 3.3.1. Blinded Pre-Validation of M3 in Samples with Different Amounts of MSM (Set 1; Fitness for Purpose)

The marker peptide (M3) was then applied in a blinded fashion to 26 samples (set 1; Supplementary Table S2) including eight samples with defined amounts (10 and 20%) of porcine MSM (1, 3, 5, and 8 mm). The aim was to investigate whether the marker can be reliably detected and which proportions of the different types of MSM can still be detected in the samples. The results showed that all 18 negative controls were correctly classified as negative, and the samples containing 10 or 20% of 1, 3, or 5 mm MSM were correctly classified as positive (e.g., SNR of 5 mm, 10%: M3-T1: 70, M3-T2: 85, M3-T3: 156, M3-T4: 356). As expected (Figure 2), the two samples containing 8 mm MSM (10%, 20%) showed only slightly higher values for the four pMRM transitions (SNR of 8 mm, 10%: M3-T1: <3, M3-T2: 7, M3-T3: 14, M3-T4: 29; 8 mm, 20%: M3-T1: <11, M3-T2: 11, M3-T3: 26, M3-T4: 60). Therefore, they could not be classified as MSM positive, as no significant differences in intensity were observed compared to the mean of the negative controls (M3-T1: <3, M3-T2: <3, M3-T3: <3, M3-T4: 11.1 ± 5.1). In summary, it should be emphasized that the lowest SNR for all four pMRM transitions of the pro-peptide protegrin-4 in the sausage products with a proportion of 10% 1/3/5 mm MSM was several times higher than the highest SNR in the negative controls (ratio of SNR: M3-T1: 11.7, M3-T2: 17.0, M3-T3: 15.6, M3-T4: 13.2).

Overall, the sensitivity for set 1 was 75%, and the specificity was 100%.

#### 3.3.2. Blinded Validation of M3 in Industrially Produced Sausages (Set 2; Precision and Trueness)

Subsequently, the method was applied to a set of 51 industrially produced sausages (set 2, Supplementary Table S3) that not only contained different proportions of porcine MSM and porcine meat but also poultry meat and poultry MSM. Again, the testing was carried out in a blinded manner.

After unblinding, the results showed all sausages with porcine MSM were correctly classified as positive and all 43 negative controls as negative, resulting in an overall sensitivity of 100% and a specificity of 100% for the assay regarding industrial samples and samples produced by local butchers.

#### 3.3.3. Evaluation of Potential Protegrin Sources Other than MSM (Fitness for Purpose, Selectivity and Sensitivity)

In addition to the validation of M3 in blinded samples, the marker was examined in three dried blood plasmas (DBPs) from different manufacturers. The aim of these investigations was to ensure that the addition of dried blood plasma did not lead to false-positive classifications because although such additives must also be declared, there is a concrete suspicion that this is not done reliably by the manufacturers (personal communication).

Traces of protegrin-4 were found in two of the three samples examined (Acontex and Foodchem; Supplementary Figure S4), showing signals for the four pMRM transitions that were found about ten times lower than in pure 8 mm MSM and several hundred times lower than in 1 mm MSM (Supplementary Table S8). In Xiam, however, the pro-peptide protegrin-4 could not be detected at all, with SNR < 3 for the four pMRM transitions (Supplementary Table S8). Dried blood plasma can therefore not be responsible for the detection of the pro-peptide protegrin-4.

#### 3.3.4. Calcium Contents of 1, 3, 5, and 8 mm MSM (Fitness for Purpose, Measurement, and Uncertainty)

In order to evaluate whether the calcium content correlates with the content of the marker M3, the calcium content was determined in the four different types of MSM (number of different samples n = 4, respectively) used in this study. We found that there was no difference in the calcium content of 1, 3, and 5 mm MSM (1 mm: 1163 ± 243 mg/kg; 3 mm: 1249 ± 613 mg/kg; 5 mm: 1241 ± 686 mg/kg), and only the 8 mm MSM had a non-significant lower level on average (600 ± 449 mg/kg).

The 3 mm MSM was the typical quality of MSM in sausage products [39]. In contrast, the 8 mm MSM was comparable to the quality of industrial minced meat (personal communication). Calcium as an indicator for MSM is therefore unsuitable because its content does not depend on the type of MSM and could lead to false-positive results, even if industrial minced meat is used.

### 3.4. Species Authentication

In addition to the identification of potential markers for porcine MSM, different tissue types from chicken, turkey, and pork were examined and evaluated together with the data collected by Wilhelm et al. [24] to search for species-specific markers for the tissue types skin and meat to enable species identification in meat products. Again, the methodology was similar to that of Wilhelm et al. [24]. After analyzing the different species samples by LC-MS/MS, the data were compiled in MarkerView V1.2.1 and sorted by tissue type for each species. The candidate markers with the lowest *p*-value were identified using ProteinPilot V5.0 (Supplementary Figures S5–S12). All candidate markers with a confirmed sequence were then tested for species specificity using Protein Blast. However, this was not possible for turkey for both skin markers (T-SK13/T-SK14) and for chicken and turkey for one meat marker each (C-ME09/T-ME12), as no sequence matches could be found in the UniProt database at the time of the study (Table 2).

Following the identification of promising candidates for species authentication, four characteristic fragment ions were defined for each marker candidate by pMRM measurements (Table 2; Supplementary Figures S13–S24).

Each tissue- and species-specific marker was then examined in all investigated tissues of the corresponding species, and the data were analyzed using MasterView^TM^. In addition, all three turkey markers without an identified sequence and the marker for chicken without sequence were also tested in each of the other two species tissue types to exclude the possibility of common markers.

All markers (two for each tissue per species; Table 2) were applied to the blinded samples of sets 1, 2, and 3 to ensure the precision of the assay. All samples (even for samples containing mixtures of different species such as chicken/turkey; Supplementary Table S4) were correctly assigned (precision 100%).

## 4. Discussion

The aim of the study was to detect porcine MSM in sausages and meat products. The methodology was comparable to a study by Wilhelm et al. [24] on poultry with improvements in the homogeneity of the crude sample. This leads to a more representative sample, resulting in highly comparable signal intensities (better repeatability) in the LC-MS/MS when the same sample was analyzed in duplicate or multiplicate.

Similar to a study in poultry [24], the initial hypothesis was that collagens from cartilage and intervertebral discs would allow detection of MSM. In contrast to poultry, it was shown in a blinded study that collagens from pork are not suitable for detecting the (undeclared) use of MSM. Nevertheless, the developed assay allows the detection of porcine MSM in sausage products by a specific marker peptide for the pro-peptide protegrin-4, which is introduced by leukocytes in the bone marrow. Furthermore, the study of processed meat and dried blood plasma showed that protegrin-4 is not introduced in higher quantities by residual blood, thus ensuring the specificity of the developed assay.

This allowed MSM to be detected with high precision in samples of unknown composition. A total of 77 unknown samples were analyzed in a blinded study, including 16 control and 8 MSM-positive samples (set 1) that were specially prepared using commercially available processed meat and MSM. This ensures the analytical validity of the sample material as required by EU Regulation 2017/625 [14]. All negative controls of set 1 were correctly classified, and only two specially prepared positive samples containing 10/20% of 8 mm porcine MSM could not be classified as positive (precision of 87.5% in the MSM group of set 1). This was expected since the structural composition of 8 mm MSM is nearly similar to industrial manufactured minced meat, and even the visual differentiation was difficult. In addition, 8 mm MSM is not usually used for falsification (personal communication), and therefore, its detection is less relevant. A further 51 samples (43 negative control and 8 MSM samples; set 2) were obtained from industry and local butchers. All negative and positive control samples of this set (precision set 2: 100%) were correctly classified, resulting in an overall precision of 93.5% for the assay in the MSM group of sets 1 + 2. Moreover, one sample from industrial production containing only 7.5% MSM in the meat content was correctly classified as positive, which means that MSM contents higher than 7.5% can be detected, assuming the declaration was correct. This limit is lower than for poultry MSM (>10%).

In addition, the study of calcium levels in the different types of MSM showed that the protegrin-4 content was not subject to the same, sometimes strong, fluctuations as the calcium content. The protegrin-4 content alone was sufficient to distinguish the four types of MSM analyzed and would therefore give a more reliable indication of the use of MSM than the calcium content. This result is supported by the fact that no further relevant sources of protegrin-4 were observed, and the protegrin-4 content, in contrast to calcium, cannot be masked or manipulated by additional processing of the MSM used for production of sausages.

It should be noted that it is currently not possible to quantify MSM in sausage products using the identified marker. Obviously, different types of MSM (1, 3, 5 and 8 mm) contribute different amounts of protegrin-4 to the food product. Therefore, only if the same amounts of MSM of the same origin were used in the production process would it be possible to draw conclusions about the type and amount of MSM. However, an increased protegrin-4 content is a reliable indication of the use of porcine MSM.

In conclusion, to our knowledge, LC-MS/MS would currently be the only alternative approach for the detection of (undeclared) porcine MSM. In addition, the method is robust and also suitable for species authenticity verification of heat-processed foods, where detection via DNA can be difficult, as DNA begins to denature at temperatures around 75 °C [40]. In the future, other methods such as infrared reflectance spectroscopy may be of interest, as Perez-Palacios et al. [41] successfully studied structural changes in pork loins due to heating. This approach seems interesting, as Wieja et al. [42] already used Raman spectroscopy to assess product quality in samples containing MSM from poultry.

An assay has also been developed to differentiate between the species chicken, turkey, and pig using two tissue types: skin and muscle meat. Species identification is particularly important in view of the prohibition of pork consumption in the Islamic and Jewish faiths (halal and kosher food). These people make up around 23% of the world’s population, and religious testing does not accept technical limits and requires sensitive tests. However, pork in particular is often added to poultry sausages to improve consistency and taste, and in 2010, the Consumer organization Foodwatch criticized the inadequate declaration of pork in poultry sausages by several manufacturers [43].

Two markers were defined for each tissue type and species and validated in a blinded study with a total of 182 samples. It should be noted that despite very good fragment spectra, no sequences could be assigned for 4 of the 12 defined potential markers. The reason for this problem was the insufficient amount of MS/MS data in the SwissProt database, especially for turkey (three out of four markers without sequence). Nevertheless, all samples were correctly classified (100% accuracy), confirming the quality and accuracy of the defined markers. Furthermore, the combination of different pMRMs in a single measurement could be used to detect not only MSM and the declared species but also substitutes, whether unintentionally or intentionally supplied, such as in the 2013 horsemeat scandal in Germany. Finally, the method offers the possibility of detecting trace amounts that may result from contaminated equipment used to process meat of different species without intermediate cleaning. For example, three poultry sausages from set 1 were found to contain trace amounts of pork due to the previous production of pork sausages. This finding was confirmed by the butchers who produced the samples (personal communication). Similarly, traces of undeclared turkey meat (less than 5%) were found in several of the purchased chicken sausages, which also indicate contamination of the equipment, which may not comply with hygiene regulations, as the use of poultry meat is subject to stricter regulations due to the risk of salmonella [44].

## 5. Conclusions

The present work demonstrates for the first time that LC-MS/MS is a reliable tool for the detection of porcine MSM in meat and sausage products. Bone marrow protegrin-4 was identified as an indicator of unlabeled MSM and allowed the detection of porcine MSM contents above 7.5% in a blinded study with 77 samples of different composition with a precision of 93.5%. In addition, biomarkers were identified to ensure the authenticity of the samples used. It was possible to define specific peptides that allow the unambiguous identification of tissue types such as skin and muscle meat from chicken, turkey, and pork with a precision of 100%.

The validity of the assay was ensured by addressing quality parameters such as accuracy, influence of matrix and concentration (fitness for purpose), limit of determination, precision, repeatability, sensitivity, and specificity. In addition, the analytical validity of the sample material was ensured by using processed meat in specially prepared samples or samples from regional butchers and industrial sources, and the assay has recently been successfully transferred to a second laboratory using a tripleQuad 5500 (AB Sciex; reproducibility).

In the future, the quantitative assays for pork protegrin-4 and poultry collagen type II alpha 1 will be developed as well as a test to quantify traces of undeclared species. Furthermore, authenticity markers for beef, sheep, and goat meat will be established.

## 6. Patents

S. Wittke and M. Hofsommer are the co-inventors of a patent regarding the detection of MSM in poultry. Since the article refers to the publication by Wilhelm et al. from 2022 [24], the respective patent applications are noticed.

German patent: Verfahren und Vorrichtung zum Nachweis von Schlachtnebenprodukten und/oder Separatorenfleisch) by the University of Applied Sciences Bremerhaven and GfL in Germany on 10 February 2020. The file number is 10 2020 103 267.6. In the meantime, the complete IP rights were transferred to the GfL in July 2024.

European patent: The European patent application EP3862754 entitled “Method for detecting slaughter byproducts and/or mechanically separated meat” was granted on 28 February 2025.

Regarding the detection of porcine MSM, no further patent application is pending or planned.

## Figures and Tables

**Table 1:** Specific peptides for MSM and related parameters like peptide sequence, retention time, m/z-value, charge state, collision energy (CE), and four pMRM transitions (T1–T4: m/z value, fragment ion, and charge state).

	Sequence Marker Peptide (Target)	Retention Time [min]	Precursor Ion [m/z]	CE	pMRM Transition No. (Product Ion, Charge State)			
			(Charge State)	[V]	T1	T2	T3	T4
**M1**	No sequence database match	3.5	477.280 (+1)	24	136.071 (+1)	199.142 (+1)	279.136 (+1)	362.213 (+1)
**M2**	No sequence database match	7.5	543.290 (+1)	26	217.120 (+1)	318.184 (+1)	364.192 (+1)	415.243 (+1)
**M3**	LDQPPKADEDPGTPKP	3.8	568.953 (+3)	24	298.674 (y6 +2)	357.177 (b3 +1)	596.340 (y6 +1)	674.841 (y13 +2)

**Table 2:** Species-specific peptides for skin (SK) and meat (ME) in chicken (C), turkey (T), and pig (P) and related parameters such as peptide sequence, retention time, m/z-value, charge state, collision energy (CE), and four pMRM transitions (m/z value, fragment ion and charge state).

	Sequence Marker Peptide (Target)	Retention Time [min]	Precursor Ion [m/z] (Charge State)	CE [V]	pMRM Transition No. (Product Ion, Charge State)			
					1	2	3	4
**C-SK04**	VGPAGPIGSRGPSG PP[Oxi]GPDGNKGE P[Oxi]GN	6.7	819.730 (+3)	40	303.130 (y3 +1)	354.210 (a6 +1)	382.209 (b5 +1)	767.700 (y25 +3)
**C-SK11**	VAVPGPMGPAGPR GLP[Oxi]GPP[Oxi]G AP[Oxi]GPQG	11.0	779.399 (+3)	32	471.220 (y5 +1)	798.906 (+2)	883.944 (+2)	933.495 (b21 +2)
**T-SK13**	No sequence database match	3.8	545.617 (+3)	20	147.076 (+1)	493.583 (+3)	646.822 (+2)	744.883 (+2)
**T-SK14**	No sequence database match	1.6	453.730 (+2)	20	228.135 (+1)	299.171 (+1)	608.278 (+1)	736.337 (+1)
**P-SK09**	VGPAGKEGPAGLP [Oxi]G	8.8	611.825 (+2)	30	533.780 (y12 +2)	878.480 (+1)	921.479 (b11 +1)	1034.563(b12 +1)
**P-SK10**	VAGAP[Oxi]GLP[Oxi] GPRG IP[Oxi]GPAG	10.1	794.926 (+2)	34	645.844 (14 +2)	1007.527 (y11 +1)	1175.653 (b13 +1)	1290.680 (y14 +1)
**C-ME06**	LLPAPGSPYGRA	8.8	599.833 (+2)	28	402.704 (y8 +2)	486.749 (y10 +2)	904.400 (y6 +1)	972.490 (y10 +1)
**C-ME09**	No sequence database match	2.9	655.285 (+2)	27	588.771 (+2)	646.281 (+2)	900.398 (+1)	1047.453 (+1)
**T-ME05**	LGQNPTNAEMNK	4.9	658.817 (+2)	30	299.171 (b3 +1)	413.214 (b4 +1)	904.419 (y8 +1)	1018.462 (y9 +1)
**T-ME12**	No sequence database match	8.4	583.628 (+3)	22	215.135 (+1)	443.261 (+1)	753.873 (+2)	818.394 (+2)
**P-ME16**	IKWGDAGATY	6.9	541.270 (+2)	23	283.129 (y2 +1)	799.410 (b8 +1)	840.352 (y8 +1)	900.457 (b9 +1)
**P-ME17**	FDQDDWKT	5.2	527.727 (+2)	22	263.103 (b2 +1)	434.240 (y3 +1)	792.352 (y6 +1)	907.379 (y7 +1)

## Data Availability

All information for sample handling/-processing and measuring conditions are included in the manuscript or in the supplements. The raw data presented in this study are available on request from the corresponding author only due to IP right issues. Further inquiries can be directed to the corresponding author. The raw data supporting the conclusions of this article will be made available by the authors on request.

## Supplementary Materials

The following supporting information can be downloaded at https://www.mdpi.com/article/10.3390/foods14081317/s1. Figure S1: Processing of the animal material; Figure S2: PCA analysis of initial subset of potential marker ions; Figure S3: MSMS spectrum for identification of the marker peptide M3; Figure S4: MSMS spectrum for the marker peptide M3 in DBP (Acontex); Figure S5: MSMS spectrum for identification of the marker peptide C-ME06; Figure S6: MSMS spectrum for identification of the marker peptide C-SK04; Figure S7: MSMS spectrum for identification of the marker peptide C-SK11; Figure S8: MSMS spectrum for identification of the marker peptide T-ME05; Figure S9: MSMS spectrum for identification of the marker peptide P-ME16; Figure S10: MSMS spectrum for identification of the marker peptide P-ME17; Figure S11: MSMS spectrum for identification of the marker peptide P-SK09; Figure S12: MSMS spectrum for identification of the marker peptide P-SK10; Figure S13: MSMS spectrum for the marker peptide C-ME06 with optimized CE showing the pMRM transitions; Figure S14: MSMS spectrum for the marker peptide C-ME09 with optimized CE showing the pMRM transitions; Figure S15: MSMS spectrum for the marker peptide C-SK04 with optimized CE showing the pMRM transitions; Figure S16: MSMS spectrum for the marker peptide C-SK11 with optimized CE showing the pMRM transitions; Figure S17: MSMS spectrum for the marker peptide T-ME05 with optimized CE showing the pMRM transitions; Figure S18: MSMS spectrum for the marker peptide T-ME12 with optimized CE showing the pMRM transitions; Figure S19: MSMS spectrum for the marker peptide T-SK13 with optimized CE showing the pMRM transitions; Figure S20: MSMS spectrum for the marker peptide T-SK14 with optimized CE showing the pMRM transitions; Figure S21: MSMS spectrum for the marker peptide P-ME16 with optimized CE showing the pMRM transitions; Figure S22: MSMS spectrum for the marker peptide P-ME17 with optimized CE showing the pMRM transitions; Figure S23: MSMS spectrum for the marker peptide P-SK09 with optimized CE showing the pMRM transitions; Figure S24: MSMS spectrum for the marker peptide P-SK10 with optimized CE showing the pMRM transitions; Table S1: Origin of the animal material of chicken (C) and turkey (T) used in the study; Table S2: List of samples (Set 1) used for blinded validation; Table S3: List of samples from retail (Set 2) used for blinded main-validation; Table S4: List of samples from retail (Set 3) used for species validation; Table S5: MS and MS/MS parameters for IDA and pMRM experiments; Table S6: Import parameters for clustering and PCA (MarkerView); Table S7: Parameters for the classification of (blinded) samples with MasterView^TM^; Table S8: Mean intensity and mean SNR of the four pMRM transitions of the peptide marker for protegrin-4 in TBP and MSM (1 mm and 8 mm).

## Abbreviations

The following abbreviations are used in this manuscript:

CE	Collision energy
cps	Counts per second
IDA	Information dependent acquisition
kDa	Kilodalton
LC	Liquid chromatography
m/z	Mass per charge ratio
MS	Mass spectrometry
MSM	Mechanically separated meat
MS/MS	Tandem mass spectrometry
(p)MRM	(Pseudo-)multiple-reaction monitoring
RT	Retention time
SNR	Signal to noise ratio
TOF	Time of flight
