# Supplementary material for: Detection of Mechanically Separated Meat from Pork in Meat-Containing Foods by Targeted LC-MS/MS Analysis
Source: Foods. 2025 Apr 10;14(8):1317. doi: 10.3390/foods14081317 (PMC12026594; doi:10.3390/foods14081317)
Supplement: Supplementary file 1 [file foods-14-01317-s001.zip › foods-3557984-Supplementary tables.pdf]

Supplementary Table S1: Origin of the animal material of chicken (C) and turkey (T) used in the study.

Sample	Designation	Origin	Agricultural business
**H01**	"Landjunker Ganzes Hähnchen"	DE BY 21057 EG	Donautal Geflügelspezialitäten, Lohmann & Co. AG, Hofweinzier 20, D-94327 Bogen
**H02**	"Landjunker Ganzes Hähnchen"	DE BY 21057 EG	Donautal Geflügelspezialitäten, Lohmann & Co. AG, Hofweinzier 20, D-94327 Bogen
**H03**	"Landjunker Ganzes Hähnchen"	DE BY 21057 EG	Donautal Geflügelspezialitäten, Lohmann & Co. AG, Hofweinzier 20, D-94327 Bogen
**H04**	"Landjunker Ganzes Hähnchen"	DE BY 21057 EG	Donautal Geflügelspezialitäten, Lohmann & Co. AG, Hofweinzier 20, D-94327 Bogen
**H05**	"Landjunker Ganzes Hähnchen"	DE BY 21057 EG	Donautal Geflügelspezialitäten, Lohmann & Co. AG, Hofweinzier 20, D-94327 Bogen
**H06**	"Wiesenhof Deutsches Geflügel Hähnchen"	unk.	Carsten Freytag Landwirt, Brokeloher Dorfst. 15, 31628 Landsbergen, Niedersachsen
**H07**	"Wiesenhof Deutsches Geflügel Hähnchen"	unk.	Carsten Freytag Landwirt, Brokeloher Dorfst. 15, 31628 Landsbergen, Niedersachsen
**H08**	"Wiesenhof Deutsches Geflügel Hähnchen"	unk.	Gerd Bröring, Essen
**H09**	"Wiesenhof Deutsches Geflügel Hähnchen"	unk.	Lütke Steinkamp Gbr, Rahden
**H10**	"Wiesenhof Deutsches Geflügel Hähnchen"	unk.	Lütke Steinkamp Gbr, Rahden
**H11**	"Landjunker Selection Maispoularde"	AT 40840 EG	Hubers Landhendl GmbH, Hauptstraße 80, AT-5223 Pfaffstätt
**H12**	"Landjunker Selection Maispoularde"	AT 40840 EG	Hubers Landhendl GmbH, Hauptstraße 80, AT-5223 Pfaffstätt
**H13**	"Landjunker Selection Maispoularde"	AT 40840 EG	Hubers Landhendl GmbH, Hauptstraße 80, AT-5223 Pfaffstätt
**H14**	"Deutsches Brathähnchen", frozen	DE ESG 253 EG	Sprehe Geflügel- u. Tiefkühlfeinkost GmbH & Co. KG, Heinrich-Beckermann-Str. 8, D-49692 Cappeln
**H15**	"Deutsches Brathähnchen", frozen	DE ESG 253 EG	Sprehe Geflügel- u. Tiefkühlfeinkost GmbH & Co. KG, Heinrich-Beckermann-Str. 8, D-49692 Cappeln
**H16**	"Deutsches Brathähnchen", frozen	DE ESG 253 EG	Sprehe Geflügel- u. Tiefkühlfeinkost GmbH & Co. KG, Heinrich-Beckermann-Str. 8, D-49692 Cappeln
**H17**	Kikok corn chicken	DE NW 20008 EG	H. Borgmeier GmbH & Co. KG, Schöninger Straße 33, D-33129 Delbrück
**H18**	Kikok corn chicken	DE NW 20008 EG	H. Borgmeier GmbH & Co. KG, Schöninger Straße 33, D-33129 Delbrück
**H19**	Kikok corn chicken	DE NW 20008 EG	H. Borgmeier GmbH & Co. KG, Schöninger Straße 33, D-33129 Delbrück
**H20**	Meat soup chicken	unk.	unk.
**H21**	Soup chicken	unk.	unk.
**H22**	"Landjunker Ganzes Hähnchen"	DE BY 21057 EG	Donautal Geflügelspezialitäten, Lohmann & Co. AG, Hofweinzier 20, D-94327 Bogen
**H23**	"Landjunker Ganzes Hähnchen"	DE BY 21057 EG	Donautal Geflügelspezialitäten, Lohmann & Co. AG, Hofweinzier 20, D-94327 Bogen
**H25**	"Landjunker Selection Maispoularde"	AT 40840 EG	Hubers Landhendl GmbH, Hauptstraße 80, AT-5223 Pfaffstätt
**H26**	"Landjunker Selection Maispoularde"	AT 40840 EG	Hubers Landhendl GmbH, Hauptstraße 80, AT-5223 Pfaffstätt
**H27**	"Landjunker Selection Maispoularde"	AT 40840 EG	Hubers Landhendl GmbH, Hauptstraße 80, AT-5223 Pfaffstätt

**H28**	Chicken thighs with back piece	DE NI 11101 EG	GEKA frisch + frost Handels GmBH & Co.KG, Paul-Wesjohann-Straße 45, 49429 Visbek
**H29**	Chicken thighs with back piece	DE NI 11101 EG	GEKA frisch + frost Handels GmBH & Co.KG, Paul-Wesjohann-Straße 45, 49429 Visbek
**H30**	Chicken thighs with back piece	DE NI 11101 EG	GEKA frisch + frost Handels GmBH & Co.KG, Paul-Wesjohann-Straße 45, 49429 Visbek
**H31**	Chicken thighs with back piece	DE NI 10021 EG	Landgeflügel FG Vertriebsgesellschaft mbH, Im Industriepark 1, 49733 Haren
**H32**	Chicken thighs with back piece	DE NI 10021 EG	Landgeflügel FG Vertriebsgesellschaft mbH, Im Industriepark 1, 49733 Haren
**H33**	Chicken thighs with back piece	DE NI 10021 EG	Landgeflügel FG Vertriebsgesellschaft mbH, Im Industriepark 1, 49733 Haren
**H34**	Chicken thighs with back piece	DE NI 10021 EG	Landgeflügel FG Vertriebsgesellschaft mbH, Im Industriepark 1, 49733 Haren
**H35**	Chicken thighs with back piece	DE NI 11101 EG	GEKA frisch + frost Handels GmBH & Co.KG, Paul-Wesjohann-Straße 45, 49429 Visbek
**H36**	Chicken thighs with back piece	DE NI 11101 EG	GEKA frisch + frost Handels GmBH & Co.KG, Paul-Wesjohann-Straße 45, 49429 Visbek
**H37**	Chicken thighs with back piece	DE NI 11101 EG	GEKA frisch + frost Handels GmBH & Co.KG, Paul-Wesjohann-Straße 45, 49429 Visbek
**H40**	"Hubers Maispoularde"	AT 40840 EG	Hubers Landhendl GmbH, Hauptstraße 80, AT-5223 Pfaffstätt
**H41**	"Hubers Maispoularde"	AT 40840 EG	Hubers Landhendl GmbH, Hauptstraße 80, AT-5223 Pfaffstätt
**H42**	Corn chicken	FR 53.121.001 CE	Galifrance, 29000 Quimper, Frankreich
**H43**	"Unser Heidegold Deutsches Hähnchen"	DE NI 11101 EG	Oldenburger GeflügelSP. GmbH & Co, 49393 Lohne
**H44**	Corn chicken	FR 53.121.001 CE	Galifrance, 29000 Quimper, Frankreich
**H45**	Corn chicken	FR 53.121.001 CE	Galifrance, 29000 Quimper, Frankreich
**H46**	"Landjunker Selection Maispoularde"	AT 40840 EG	Hubers Landhendl GmbH, Hauptstraße 80, AT-5223 Pfaffstätt
**H47**	"Fair & Gut Frisches Hähnchen"	DE NI 11101 EG	GEKA frisch + frost Handels GmBH & Co.KG, Paul-Wesjohann-Straße 45, 49429 Visbek
**H48**	"Fair & Gut Frisches Hähnchen"	DE NI 11101 EG	GEKA frisch + frost Handels GmBH & Co.KG, Paul-Wesjohann-Straße 45, 49429 Visbek
**T01**	"Landjunker Putenunterkeulen"	DE NI 10321 EG	Frischland Premium Spezialitäten GmbH & Co. KG, Hofweinzier 20, D-94327 Bogen
**P02**	"Landjunker Putenunterkeulen"	DE NI 10321 EG	Frischland Premium Spezialitäten GmbH & Co. KG, Hofweinzier 20, D-94327 Bogen
**T03**	"Metzgerfrisch Puten-Unterkeule"	DE NI 10321 EG	Frischland Premium Spezialitäten GmbH & Co. KG, Hofweinzier 20, D-94327 Bogen
**T04**	"Metzgerfrisch Puten-Unterkeule"	DE NI 10321 EG	Frischland Premium Spezialitäten GmbH & Co. KG, Hofweinzier 20, D-94327 Bogen
**T05**	"Metzgerfrisch Puten-Unterkeule"	DE NI 10321 EG	Frischland Premium Spezialitäten GmbH & Co. KG, Hofweinzier 20, D-94327 Bogen
**T06**	"Metzgerfrisch Puten-Unterkeule"	DE NI 10321 EG	Frischland Premium Spezialitäten GmbH & Co. KG, Hofweinzier 20, D-94327 Bogen
**T07**	"meine Metzgerei Putenoberkeule"	DE NI 10321 EG	GEKA frisch + frost Handels GmbH & Co. KG, Paul-Wesjohann-Straße 45, D-49429 Visbek
**T08**	"meine Metzgerei Putenoberkeule"	DE NI 10321 EG	GEKA frisch + frost Handels GmbH & Co. KG, Paul-Wesjohann-Straße 45, D-49429 Visbek
**T09**	"Heidemark Putenschenkel natur"	DE NI 10067 EG	Heidemark GmbH Lether Gewerbestraße 2, D-26197 Ahlhorn
**T10**	"Heidemark Putenschenkel natur"	DE NI 10067 EG	Heidemark GmbH Lether Gewerbestraße 2, D-26197 Ahlhorn
**T11**	"Purland Qualitätsfleisch Putenunterkeule"	DE NI 10067 EG	Heidemark GmbH Lether Gewerbestraße 2, D-26197 Ahlhorn

**T12**	"Purland Qualitätsfleisch Putenunterkeule"	DE NI 10067 EG	Heidemark GmbH Lether Gewerbestraße 2, D-26197 Ahlhorn
**T21**	Turkey carcass	DE NW-20123 EG	Bartels GmbH & Co.KG, Langenberger Str. 125, 33397 Mastholte
**T22**	Turkey carcass	DE NW-20123 EG	Bartels GmbH & Co.KG, Langenberger Str. 125, 33397 Mastholte
**T23**	Turkey carcass	DE NW-20123 EG	Bartels GmbH & Co.KG, Langenberger Str. 125, 33397 Mastholte
**T24**	Turkey carcass	DE NW-20123 EG	Bartels GmbH & Co.KG, Langenberger Str. 125, 33397 Mastholte

Supplementary Table S2: List of samples (Set 1) used for blinded validation. **NC:** Negative control samples (no MSM) produced with pork and poultry; **PC:** Positive control samples containing 10 and 20 % of the four investigated types of MSM (1, 3, 5 and 8 mm) from pork.

Sample	Designation	Species/MSM content
NC1-01	Fleischwurst (Rindfleisch, Schweinefleisch, Rückenspeck)	beef, pork
NC1-02	Wiener Würstchen (Schweinefleisch, Speck)	pork
NC1-03	Wiener Würstchen (Rindfleisch, Schweinefleisch, Deckelfett)	beef, pork
NC1-04	Geflügelfleischwurst (Putenverarbeitungsfleisch, Hühnerhaut)	turkey, chicken
NC1-05	Fleischwurst im Ring (Schweinefleisch, Rückenspeck, Deckelfett vom Schinken)	pork
NC1-06	Pommersche feine Brühwurst, Roh (Schweinefleisch, Schweinefleisch, Kalbfleisch)	beef, pork
NC1-07	Brühwurst zum Rösten (Verarbeitungsfleisch Schwein, Schweinespeck)	pork
NC1-08	Truthahn Lyoner glatt, ohne Zugabe (Pute Brustabschnitte, Hühnerhaut)	turkey, chicken
NC1-09	Truthahn Bierschinken (Pute Verarbeitungsfleisch, Hühnerhaut,)	turkey, chicken
NC1-10	Bockwurst (Schweinefleisch, Speck)	pork
NC1-11	Rost Bratwurst (Schweinefleisch)	pork
NC1-12	Bierschinken geschnitten (Schweinefleisch, Deckelfett)	pork
NC1-13	Truthahn Jagdwurst (Truthahn, Hühnerhaut)	turkey, chicken
NC1-14	Bierschinken Grundbrät (Schweinefleisch, Rückenspeck, grobe Einlage aus Schweinegulasch)	pork
NC1-15	Jagdwurst (Schweinefleisch, Speck)	pork
NC1-16	Fleischbrät (Rindfleisch, Kopffleisch gekocht)	beef, pork
NC1-17	Truthahn Lyoner mit Petersilie (Putenfleisch, Hühnerhaut)	turkey, chicken
NC1-18	Truthahn Paprika Lyoner (Putenfleisch, Hühnerhaut)	turkey, chicken
PC1-01	Fleischbrät (Rindfleisch, Kopffleisch gekocht, Schweinefleisch, MSM (1mm) 10%)	beef, pork, MSM pork 10 % (1 mm)
PC1-02	Fleischbrät (Rindfleisch, Kopffleisch gekocht, Schweinefleisch, MSM (5mm) 10%)	beef, pork, MSM pork 10 % (5 mm)

PC1-03	Fleischbrät (Rindfleisch, Kopffleisch gekocht, Schweinefleisch, MSM (3mm) 10%)	beef, pork, MSM pork 10 % (3 mm)
PC1-04	Fleischbrät (Rindfleisch, Kopffleisch gekocht, Schweinefleisch, MSM (3mm) 20%)	beef, pork, MSM pork 20 % (3 mm)
PC1-05	Fleischbrät (Rindfleisch, Kopffleisch gekocht, Schweinefleisch, MSM (5mm) 20%)	beef, pork, MSM pork 20 % (5 mm)
PC1-06	Fleischbrät (Rindfleisch, Kopffleisch gekocht, Schweinefleisch, MSM (1mm) 20%)	beef, pork, MSM pork 20 % (1 mm)
PC1-07	Fleischbrät (Rindfleisch, Kopffleisch gekocht, Schweinefleisch, MSM (8mm) 20%)	beef, pork, MSM pork 20 % (8 mm)
PC1-08	Fleischbrät (Rindfleisch, Kopffleisch gekocht, Schweinefleisch, SMSM (8mm) 10%)	beef, pork, MSM pork 10 % (8 mm)

Supplementary Table S3: List of samples from retail (Set 2) used for blinded main-validation. **NC:** Negative control samples (no pork MSM) produced with pork and poultry; **PC:** Positive control samples with three samples containing 7.5, 10 and 20 % and five samples with unknown proportions of MSM from pork.

Sample	Designation	Species/MSM content
NC2-01	Fulya Hindi Salam - Putenwurst	turkey, chicken
NC2-02	Schröder's Bio Geflügel-Fleischwurst	poultry
NC2-03	Fleischwurst	pork
NC2-04	Wiener Würstchen	pork
NC2-05	Lyoner	pork
NC2-06	MOR Semja Brotaufstrich mit Putenseparatorenfleisch	chicken, turkey, MSM chicken 10 %, MSM turkey 10 %
NC2-07	MOR Semja Brotaufstrich mit Putenseparatorenfleisch	chicken, turkey, MSM chicken 10 %, MSM turkey 10 %
NC2-08	Profi Pasztet z indykiem pelen smaku	turkey, MSM turkey 14 %
NC2-09	Fleischwurst	pork
NC2-10	Brüh Würstchen	pork
NC2-11	Lyoner	pork
NC2-12	Fleischwurst	pork
NC2-13	Brüh Würstchen	pork
NC2-14	Lyoner	pork
NC2-15	Fleischwurst	pork
NC2-16	Brüh Würsten	pork
NC2-17	Lyoner	pork
NC2-18	Fleischwurst	pork
NC2-19	Brüh Würstchen	pork

NC2-20	Lyoner	pork
NC2-21	Fleischwurst	pork
NC2-22	Brüh Würstchen	pork
NC2-23	Lyoner	pork
NC2-24	Fleischwurst	pork
NC2-25	Brüh Würstchen	pork
NC2-26	Lyoner	pork
NC2-27	Fleischwurst	pork
NC2-28	Brüh Würstchen	pork
NC2-29	Wiener Würstchen	pork
NC2-30	Putenbratwurst	turkey
NC2-31	Puten Wiener	turkey
NC2-32	Geflügel Mortadella	turkey, chicken
NC2-33	Geflügel Bratwurst	turkey, chicken
NC2-34	Jagdwurst	turkey
NC2-35	Mortadella	turkey
NC2-36	Wiener Würstchen	turkey
NC2-37	Geflügelfleisch Käse ofengeb.	poultry
NC2-38	Puten Lyoner	turkey
NC2-39	Mortadella z papryka	MSM turkey 11 %, MSM chicken 44 %
NC2-40	Dobrowit Pasztet z drobiem	pork, chicken, MSM chicken 7.5 %
NC2-41	Pekpol Mortadela	pork, chicken, MSM chicken 16.9 %
NC2-42	Drosed Parowki drobiowe	MSM chicken 47 %
NC2-43	Stol Soltysa Parowki Drobiowe	MSM chicken 55 %
PC2-01	Gratka Tyrolese Tinned Meat	pork, MSM pork
PC2-02	Pasztetowa Wieprzowa	pork, MSM pork
PC2-03	Schweinefleisch-Pastete	pork, MSM pork 7.5 %
PC2-04	7ja Schweinefleisch mit Separatoren	pork, MSM pork 15 %
PC2-05	EvraMeat Meat Luncheon	MSM pork, MSM chicken
PC2-06	EvraMeat Mielonka Miesna	MSM pork, MSM chicken

PC2-07	unox SMAC de enige echte	MSM pork 20 %, MSM chicken 31 %
PC2-08	Duda pasztetowa firmow	pork, MSM pork, chicken

Supplementary Table S4: List of samples from retail (Set 3) used for species validation.

Sample	Designation	Species
S3-01	Freiländer Bio Geflügel Puten Aufschnitt nach Saftschinken Art	turkey
S3-02	echt und recht Bio Geflügel Salami	turkey, chicken
S3-03	Fleischwurst vom Ring	beef, pork
S3-04	Bierschinken	pork
S3-05	Gelbwurst	pork
S3-06	Gelbwurst am Stück	pork
S3-07	Lyoner	pork
S3-08	Bierschinken	pork
S3-09	Leberwurst	pork
S3-10	Lyoner	pork
S3-11	Gelbwurst	pork
S3-12	Breit Fleischwurst	pork
S3-13	Jagdwurst	pork
S3-14	Gelbwurst	pork
S3-15	Breit Fleischwurst	pork
S3-16	Bierschinken	pork
S3-17	Gelbwurst	pork
S3-18	Schinkenwurst Paprika	pork
S3-19	Schinkenwurst Champignons	pork
S3-20	Lyoner	beef, pork
S3-21	Puten Wiener	turkey
S3-22	Fleischwurst	turkey
S3-23	Putensalami	turkey
S3-24	Putenlyoner	turkey

S3-25	Bierschinken	pork
S3-26	Brühwurstpastete	turkey, chicken
S3-27	Truthahnsalami	turkey
S3-28	Geflügel Wiener	turkey, chicken
S3-29	Geflügel Salami	pork, turkey
S3-30	Geflügel Mortadella	turkey, chicken
S3-31	Putenleberrolle	turkey
S3-32	Gavrilovic Aufstrich mit Putenleber	turkey, MSM turkey 50 %
S3-33	Schröder's Bio Geflügel-Bratwurst m. Bärlauch	turkey, chicken
S3-34	NEULAND Putenbratwurst	turkey
S3-35	Schröder's Bio Geflügel-Lyoner aus Hähnchenfleisch	turkey, chicken
S3-36	NEULAND Putenfleischkäse aus 100% Putenfleisch	turkey
S3-37	NEULAND Putensalami 100% Pute	turkey
S3-38	Schröder's Bio Geflügel-Fleischwurst	turkey, chicken
S3-39	7ja Schweinefleisch mit Separatoren	pork, MSM pork 15 %
S3-40	Profi Pasztet z indykiem pelen smaku	turkey, MSM turkey 14 %
S3-41	Fleischwurst	beef, pork, chicken
S3-42	Gratka Tyrolese Tinned Meat	pork, MSM pork
S3-43	Gavrilovic Aufstrich mit Putenleber	turkey, MSM turkey 50 %
S3-44	Truthahnsalami	turkey
S3-45	Pasztetowa Wieprzowa	pork, MSM pork
S3-46	unox SMAC de enige echte	MSM pork 20 %, MSM chicken 31 %
S3-47	Geflügelbratwurst	turkey
S3-48	Kleine Mortadella	pork
S3-49	Fleischwurst	pork
S3-50	Brühwürstchen	pork
S3-51	NEULAND Putenbratwurst	turkey
S3-52	Fleischwurst	pork
S3-53	Feine Leberwurst	pork
S3-54	Gekochte Mettwurst	pork

S3-55	Wildschwein-Leberwurst	wild pork
S3-56	Geflügelwiener	poultry
S3-57	Grobes Streichmett	pig
S3-58	Mettwurst	pig
S3-59	Leberwurst	pig
S3-60	Jagdwurst	pig
S3-61	Bockwurst	poultry
S3-62	Bratwurst	poultry
S3-63	Leberkäse	poultry
S3-64	Fleischwurst	poultry
S3-65	Paprikalyoner	poultry
S3-66	Lyoner mit Pilzen	poultry
S3-67	Fleischwurst	pork
S3-68	Fleischwurst mit Collagenhydrolysat	pork
S3-69	Lyoner mit Collagenhydrolysat	pork
S3-70	Geflügeljagdwurst	poultry
S3-71	Geflügelbierschinken	poultry
S3-72	Wiener	pork
S3-73	Geflügellyoner	pork
S3-74	AL-RAII Halal Fleischerzeugnis aus Hühnerseparatorenfleisch	MSM chicken 79 %
S3-75	Aladin Tavuk Dilim Geflügelfleischerzeugnis mit Kartoffelstärke	MSM turkey 19 %, MSM chicken 19 %
S3-76	Sohbet Tavuk Salam Hühnerseparatorenfleischerzeugnis	MSM chicken 41 %
S3-77	Profi Pastete mit Geflügel und Schnittlauch	poultry, MSM chicken 13,5 %
S3-78	Drop Pastete mit Geflügel	poultry, MSM chicken 18 %
S3-79	Kolbassoff Hähnchenhaltiger Brotaufstrich mit Hähnchenseparatorenfleisch	pork, MSM chicken 14 %
S3-80	Brotaufstrich mit Hähnchenseparatorenfleisch	duck, chicken, MSM chicken 14 %
S3-81	Drop Super hot Brotaufstrich mit Geflügel	pork, chicken, MSM chicken 21 %
S3-82	Brotaufstrich mit Hähnchenseparatorenfleisch und Gänseseparatorenfleisch	goose, MSM chicken 14 %
S3-83	Brotaufstrich mit Hähnchenseparatorenfleisch	pork, chicken, MSM chicken 19 %
S3-84	Brotaufstrich nach Art einer polnischen Kochstreichwurst mit Soja	duck, MSM chicken 14 %

S3-85	Özyörem Jumbo Sade Sosis Hühnerseparatorenfleischerzeugnis	beef, MSM chicken 61 %
S3-86	Robert Halal Chicken Luncheon Meat Hot Spiced	MSM chicken 90 %
S3-87	Destan Biberli Salam Hühnerseparatorenfleischerzeugnis	MSM chicken 37 %
S3-88	esma Mini Sosis Hähnchenfleischerzeugnis	MSM chicken 75 %
S3-89	YAYLA Cocktail-Würstchen Mini Hühnerwürstchen	MSM chicken 55 %
S3-90	Köytad Röllchen nach Cevapcici-Art	beef, MSM chicken 21 %
S3-91	Öz Köytad Tavuk Burger Geflügel Bratling	MSM chicken 78 %
S3-92	Halal Al-RAII Fleischerzeugnis aus Hühnerseparatorenfleisch	MSM chicken 48 %
S3-93	Pavo Frankfurt Separatorenfleisch-Geflügelwurst	MSM poultry 91 %
S3-94	unknown	turkey, chicken
S3-95	unknown	turkey, chicken
S3-96	unknown	chicken
S3-97	unknown	turkey, chicken
S3-98	unknown	turkey, chicken
S3-99	unknown	turkey, chicken
S3-100	unknown	chicken
S3-101	unknown	turkey, chicken
S3-102	unknown	turkey, chicken
S3-103	unknown	turkey
S3-104	unknown	turkey, chicken
S3-105	unknown	chicken

Supplementary Table S5: MS and MS/MS parameters for IDA and pMRM-experiments; for pMRM start and end masses and CE are adapted to the marker ions, respectively.

Parameter (IDA; full scan)	MS and MS/MS values
ESI Source [V]	5200
Source Temperature [°C]	420
Curtain gas pressure [kPa]	30

Collision energy [V]	rolling
Nebulizer gas [kPa]	14
Heating gas [kPa]	45
Start mass [m/z] (IDA only)	200 / 50
End mass [m/z] (IDA only)	1500 / 1500

Supplementary Table S6: Import parameters for data compilation (clustering) and the principal component analysis (MarkerView).

Min. Retention Time	0.25 min
Max. Retention Time	20.00 min
Noise Threshold	100
Min. Spectral Peak Width	5 ppm
Min. RT Peak Width	6 scans
RT Tolerance	± 0.1 min
Mass Tolerance	± 0.005 Da

Supplementary Table S7: Parameters for the classification of (blinded) samples with MasterView™.

Do not calculate details for XIC with intensity <	50 counts
or SNR <	3

Default XIC Width (Da)	0.05
Default Retention Time Width (min)	0.2
Default Threshold (cps)	50
Default Threshold (ratio of control)	10

Supplementary Table S8: Mean intensity and mean SNR of the four pMRM-transitions of the peptide marker for protegrin-4 in TBP and MSM (1 mm and 8 mm). For assignment of the markers to all samples a difference in retention time of Δ t < ± 0.1 min and a mass accuracy of Δ m/z < ± 0.01 Da are mandatory.

	**mean intensity [cps] and SNR (in brackets) of pMRM-transition (product ion, charge state)**			
	**298.674 (y6 +2)**	**357.177 (b3 +1)**	**596.340 (y6 +1)**	**674. 841 (y13 +2)**
**Acontex**	52 (8)	73 (46)	109 (18)	193 (26)
**Foodchem**	72 (10)	82 (14)	126 (28)	236 (30)
**Xiam**	< 50 (< 3)	< 50 (< 3)	< 50 (< 3)	< 50 (< 3)
**MSM (1 mm)**	9220 (726)	10587 (738)	22418 (974)	50827 (1274)
**MSM (8 mm)**	370 (37)	441 (55)	807 (136)	1962 (285)
