# Supplementary material for: Detection of Mechanically Separated Meat from Pork in Meat-Containing Foods by Targeted LC-MS/MS Analysis
Source: Foods. 2025 Apr 10;14(8):1317. doi: 10.3390/foods14081317 (PMC12026594; doi:10.3390/foods14081317)
Supplement: Supplementary file 1 [file foods-14-01317-s001.zip › foods-3557984-Supplementary figures.pdf]

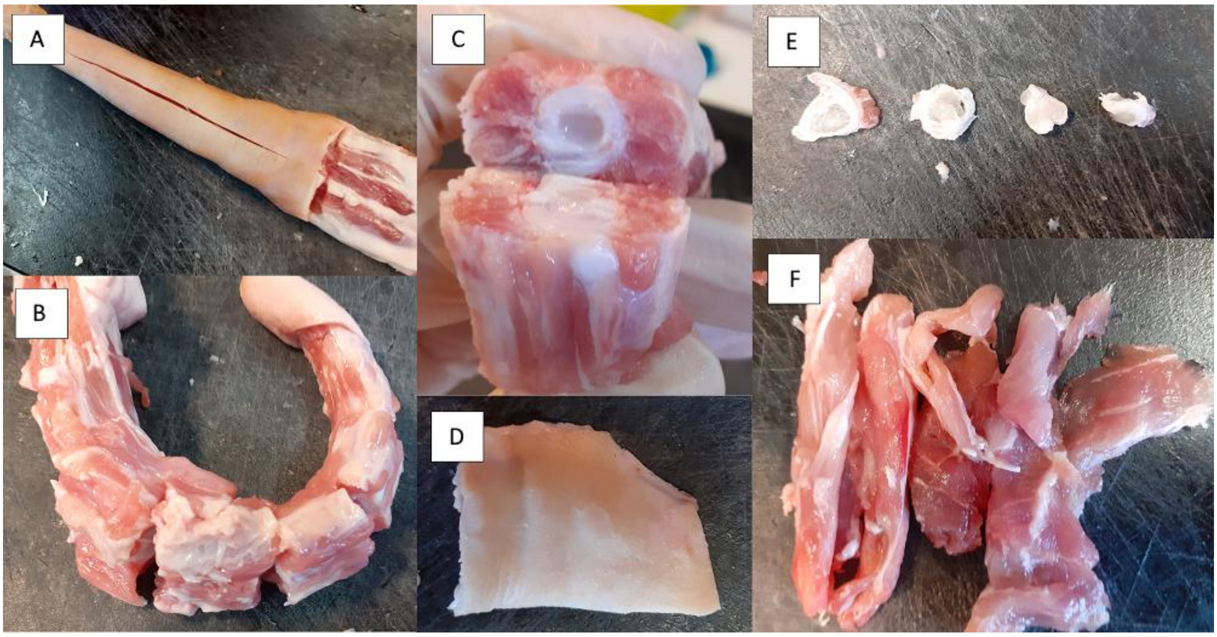

Supplementary Figure S1: Processing of the pig tails (A to C) and cutting into the individual tissue types (compartments) skin (D), intervertebral disc (E) and meat (F).

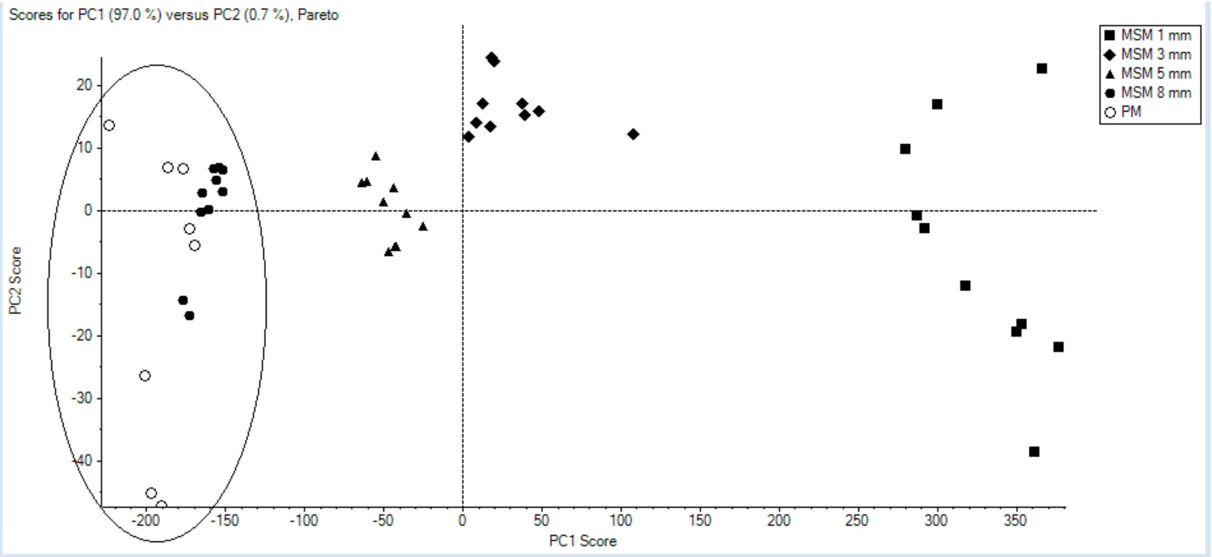

Supplementary Figure S2: Discrimination of MSM (1, 3 and 5 mm) samples from processed meat and MSM (8 mm) by PCA-analysis on the basis of an initial subset of 50 specific ions after untargeted LC-MS analysis. The processed meat and MSM (8 mm) samples are highlighted by the elliptic circle and can be distinguished from the other types of MSM by means of PCA analysis (3.1).

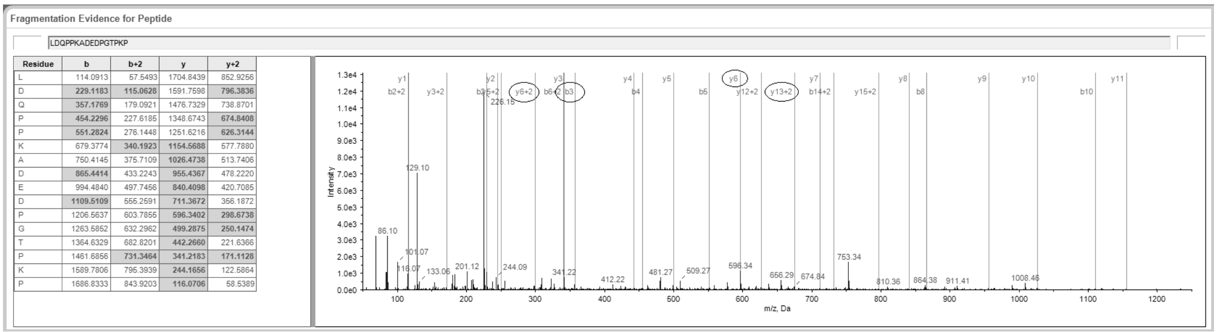
Fragmentation Evidence for Peptide

LDQPPKADEDPGTPKP

Residue	b	b+2	y	y+2
L	114.0913	57.5493	1704.8439	852.9256
D	229.1183	115.0628	1591.7598	796.3836
Q	357.1769	179.0921	1476.7329	738.8701
P	454.2296	227.6185	1348.6743	674.8408
P	551.2824	276.1448	1251.6216	626.3144
K	679.3774	340.1923	1154.5688	577.7880
A	750.4145	375.7109	1026.4738	513.7406
D	865.4414	433.2243	955.4367	478.2220
E	994.4840	497.7456	840.4098	420.7085
D	1109.5109	555.2591	711.3672	356.1872
P	1206.5637	603.7855	596.3402	298.6738
G	1263.5852	632.2962	499.2875	250.1474
T	1364.6329	682.8201	442.2660	221.6366
P	1461.6856	731.3464	341.2183	171.1128
K	1589.7806	795.3939	244.1656	122.5864
P	1686.8333	843.9203	116.0706	58.5389

Supplementary Figure S3: MSMS-spectrum including the pMRM-transitions (highlighted by circles) and sequence coverage (table; detected fragment ions are highlighted in bold) for the marker peptide M3 (LDQPPKADEDPGTPKP; m/z 568.953; 3-fold charged; UniProt: PG4_PIG) in MSM (1 mm) after thermolysin digest showing the sequence identified by ProteinPilot.

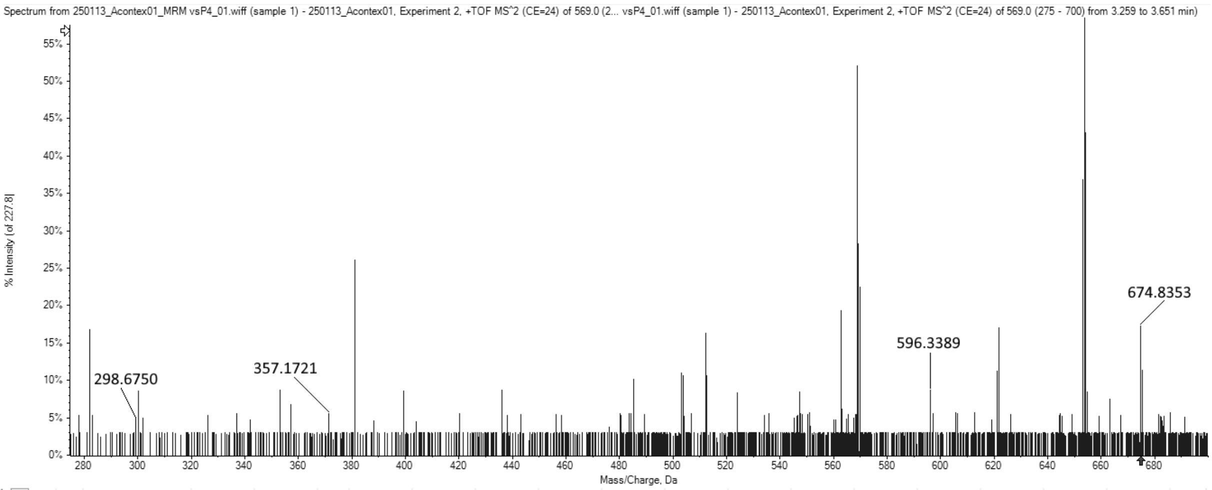

#		Mass RT Isotope Library Formula	Name	Mass (Da)	Int Std	Extraction Mass (Da)	Width (Da)	Width (ppm)	Expected RT (min)	RT Width (min)	Fragment Mass (Da)	Extraction Experiment (#)	Found At Mass (Da)	Error (mDa)	Found At RT (min)	RT Delta (min)	Intensity	Area	S:N
7	✓	✓✓●●●	y6 +2	568.953		568.953	0.05	87.881	3	1	298.674	2*	568.95764	4.6	3.06	0.06	55	5	8
8	✓	✓✓●●●	b3 +1	568.953		568.953	0.05	87.881	3	1	357.177	2*	568.95764	4.6	3.03	0.03	61	4	12
9	✓	✓✓●●●	y6 +1	568.953		568.953	0.05	87.881	3	1	596.34	2*	568.95764	4.6	3.06	0.06	128	15	24
10	✓	✓✓●●●	y13 +2	568.953		568.953	0.05	87.881	3	1	674.841	2*	568.95764	4.6	3.07	0.07	234	34	31

Supplementary Figure S4: MSMS-spectrum for the marker peptide M3 (LDQPPKADEDPGTPKP; m/z 568.953; 3-fold charged; UniProt: PG4_PIG) in DBP (Acontex) after thermolysin digest with optimized CE (24 V) showing the defined pMRM-transitions for identification (table: positive results shown by MasterView for the four pMRM-transitions).

Fragmentation Evidence for Peptide

LLPAPGSPYGRA

Residue	b	b+2	y	y+2
L	114.0913	57.5493	1198.6579	**599.8326**
L	**227.1754**	114.0913	1085.5738	**543.2905**
P	**324.2282**	162.6177	**972.4898**	**486.7485**
A	**395.2653**	198.1363	**875.4370**	**438.2221**
P	492.3180	246.6627	**804.3999**	**402.7036**
G	549.3395	275.1734	**707.3471**	354.1772
S	636.3715	318.6894	**650.3257**	325.6665
P	733.4243	367.2158	**563.2936**	**282.1504**
Y	896.4876	448.7475	**466.2409**	233.6241
G	953.5091	477.2582	**303.1775**	152.0924
R	1109.6102	555.3087	246.1561	123.5817
A	1180.6473	590.8273	90.0550	45.5311

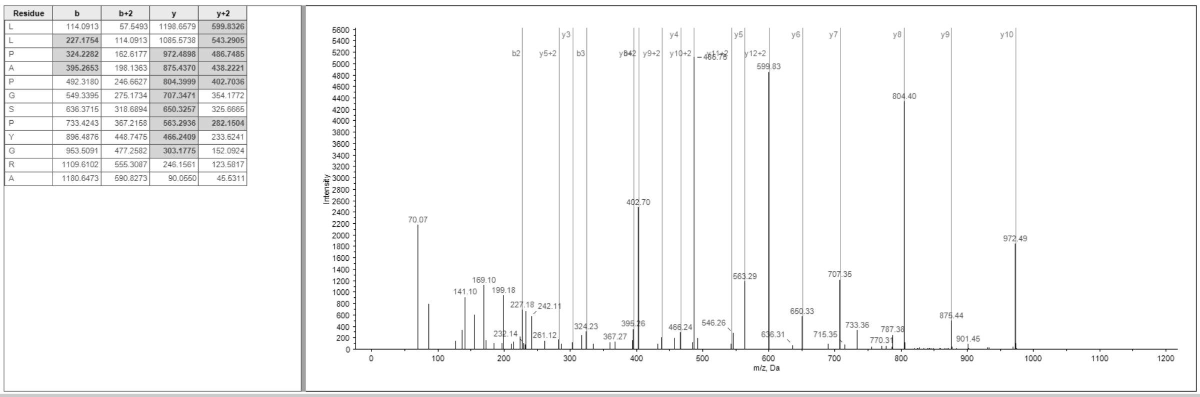

Supplementary Figure S5: MSMS-spectrum including the pMRM-transitions and sequence coverage (table; detected fragment ions are highlighted in bold) for the marker peptide C-ME06 (LLPAPGSYPYGRA; m/z 599.822; 2-fold charged; UniProt: HSPB1_CHICK) in chicken breast meat after thermolysin digest.

Fragmentation Evidence for Peptide

VGPAGPIGSRGPSGPP[Oxi]GPDGNKGEP[Oxi]GN

Residue	b	b+2	y	y+2
V	100.0757	50.5415	2457.1753	1229.0913
G	**157.0972**	79.0522	2358.1069	**1179.5571**
P	**254.1499**	127.5786	2301.0854	**1151.0464**
A	**325.1870**	163.0972	2204.0327	**1102.5200**
G	**382.2085**	191.6079	2132.9955	**1067.0014**
P	479.2613	240.1343	2075.9741	**1038.4907**
I	**592.3453**	296.6763	1978.9213	**989.9643**
G	649.3668	**325.1870**	1865.8373	**933.4223**
S	**736.3988**	368.7030	1808.8158	**904.9115**
R	**892.4999**	446.7536	1721.7838	**861.3955**
G	**949.5214**	475.2643	1565.6827	**783.3450**
P	1046.5742	523.7907	1508.6612	754.8342
S	**1133.6062**	567.3067	1411.6084	706.3079
G	**1190.6276**	595.8175	1324.5764	**662.7918**
P	1287.6804	**644.3438**	1267.5549	**634.2811**
P[Oxi]	1400.7281	700.8677	**1170.5022**	585.7547
G	1457.7496	**729.3784**	**1057.4545**	529.2309
P	1554.8023	777.9048	**1000.4330**	**500.7202**
D	1669.8293	**835.4183**	903.3803	452.1938
G	1726.8507	863.9290	**788.3533**	394.6803
N	1840.8937	920.9505	**731.3319**	366.1696
K	1968.9886	984.9979	**617.2889**	309.1481
G	2026.0101	1013.5087	**489.1940**	245.1006
E	2155.0527	**1078.0300**	**432.1725**	216.5899
P[Oxi]	2268.1003	1134.5538	**303.1299**	152.0686
G	2325.1218	1163.0645	**190.0822**	95.5448
N	2439.1647	1220.0860	**133.0608**	67.0340

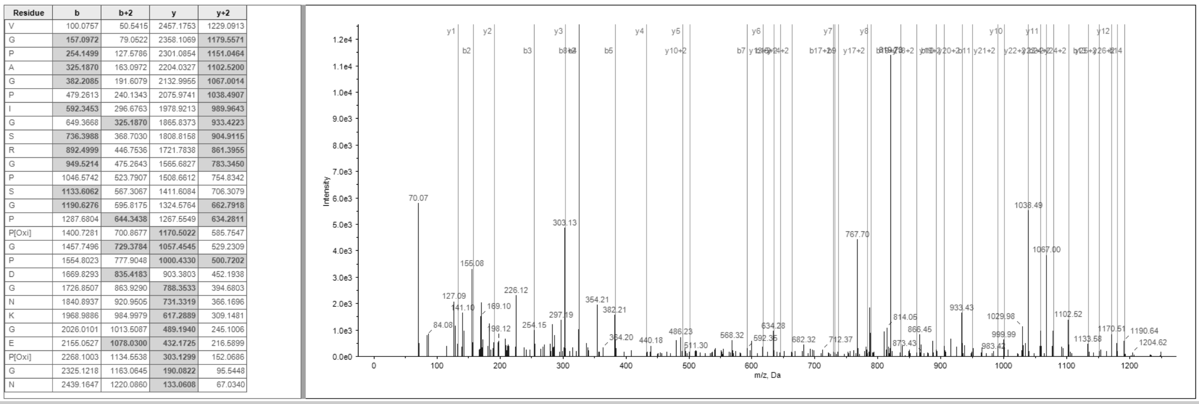

Supplementary Figure S6: MSMS-spectrum including the pMRM-transitions and sequence coverage (table; detected fragment ions are highlighted in bold) for the marker peptide C-SK04 (VGPAGPIGSRGPSGPP[Oxi]GPDGNKGEP[Oxi]GN; m/z 819.730; 3-fold charged; UniProt: CO1A2_CHICK) in chicken skin after thermolysin digest.

Fragmentation Evidence for Peptide

VAVPGPMGPAGPRGLP[Oxi]GPP[Oxi]GAP[Oxi]GPQG

Residue	b	b+2	y	y+2
V	100.0757	50.5415	2336.1816	1168.5944
A	**171.1128**	**86.0600**	2237.1132	1119.0602
V	**270.1812**	135.5942	2166.0760	**1083.5417**
P	**367.2340**	184.1206	2067.0076	**1034.0075**
G	**424.2554**	212.6314	1969.9549	**985.4811**
P	521.3082	261.1577	1912.9334	**956.9703**
M	**652.3487**	326.6780	1815.8806	908.4440
G	**709.3702**	**355.1887**	1684.8402	842.9237
P	806.4229	403.7151	1627.8187	814.4130
A	**877.4600**	439.2337	1530.7659	765.8866
G	**934.4815**	**467.7444**	1459.7288	730.3680
P	1031.5343	516.2708	1402.7074	701.8573
R	1187.6354	**594.3213**	1305.6546	653.3309
G	**1244.6568**	622.8321	1149.5535	575.2804
L	1357.7409	**679.3741**	1092.5320	546.7696
P[Oxi]	1470.7886	735.8979	**979.4480**	490.2276
G	1527.8100	**764.4087**	866.4003	433.7038
P	1624.8628	**812.9350**	**809.3788**	405.1930
P[Oxi]	1737.9105	**869.4589**	**712.3260**	356.6667
G	1794.9320	**897.9696**	599.2784	300.1428
A	1865.9691	**933.4882**	**542.2569**	271.6321
P[Oxi]	1979.0167	**990.0120**	**471.2198**	**236.1135**
G	2036.0382	**1018.5227**	**358.1721**	179.5897
P	2133.0910	**1067.0491**	**301.1506**	151.0790
Q	2261.1495	1131.0784	**204.0979**	102.5526
G	2318.1710	**1159.5891**	**76.0393**	38.5233

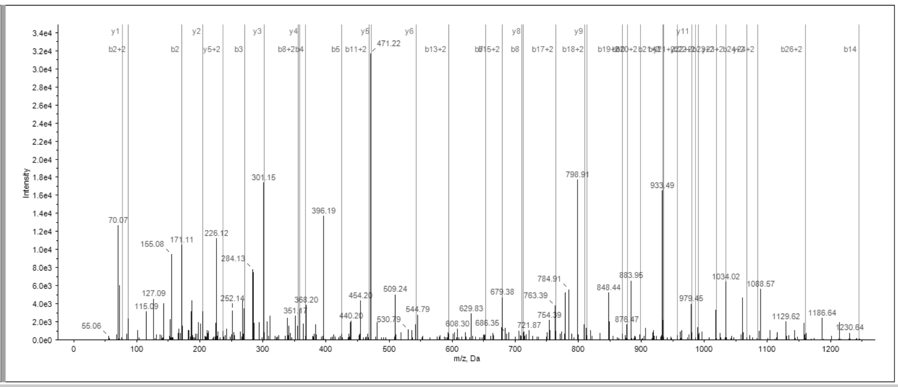

Supplementary Figure S7: MSMS-spectrum including the pMRM-transitions and sequence coverage (table; detected fragment ions are highlighted in bold) for the marker peptide C-SK11 (VAVPGPMGPAGPRGLP[Oxi]GPP[Oxi]GAP[Oxi]GPQG; m/z 779.399; 3-fold charged; UniProt: CO1A1_CHICK) in chicken skin after thermolysin digest.

Fragmentation Evidence for Peptide

LGQNPTNAEVMK[AAA]

Residue	b	b+2	y	y+2
L	114.0913	57.5493	1316.6151	**658.8112**
G	**171.1128**	86.0600	**1203.5310**	**602.2692**
Q	**299.1714**	150.0893	**1146.5096**	**573.7584**
N	**413.2143**	207.1108	**1018.4510**	**509.7291**
P	510.2671	255.6372	**904.4081**	**452.7077**
T	611.3148	306.1610	**807.3553**	404.1813
N	725.3577	363.1825	**706.3076**	353.6574
A	**796.3948**	398.7010	**592.2647**	296.6360
E	**925.4374**	463.2223	**521.2276**	261.1174
V	1024.5058	512.7565	**392.1850**	196.5961
M	1155.5463	578.2768	293.1166	147.0619
K[AAA]	1298.6045	**649.8059**	162.0761	81.5417

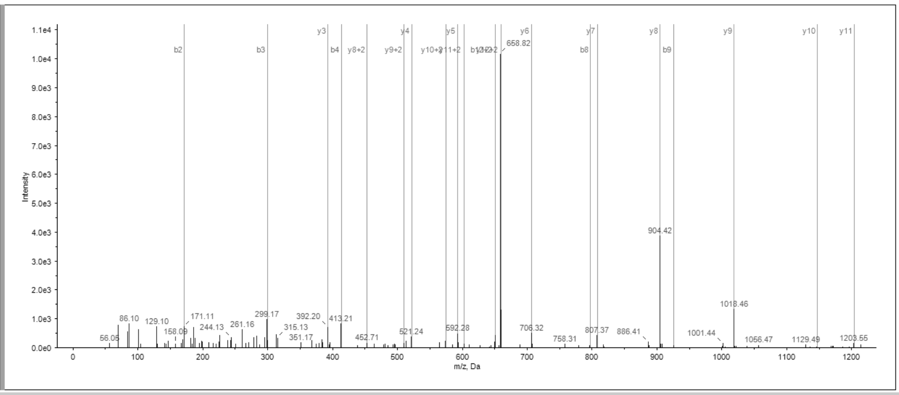

Supplementary Figure S8: MSMS-spectrum including the pMRM-transitions and sequence coverage (table; detected fragment ions are highlighted in bold) for the marker peptide T-ME05

(LGQNPTNAEMNK; m/z 658.817; 2-fold charged; UniProt: A0A8B6WYV6_MELGA) in turkey thigh after thermolysin digest.

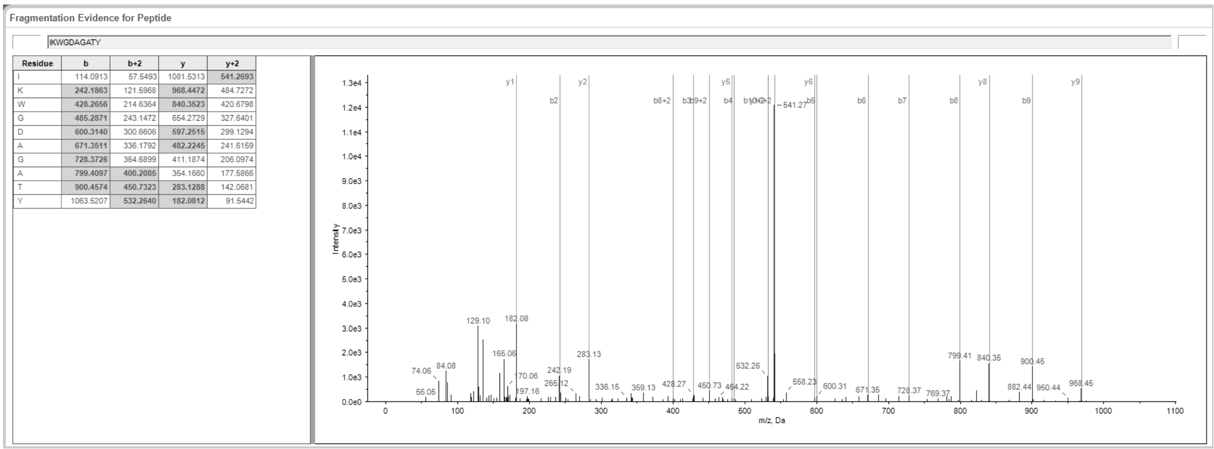

Residue	b	b+2	y	y+2
I	114.0913	57.5493	1081.5313	**541.2693**
K	**242.1863**	121.5968	**968.4472**	484.7272
W	**428.2656**	214.6364	**840.3523**	420.6798
G	**485.2871**	243.1472	654.2729	327.6401
D	**600.3140**	300.6606	**597.2515**	299.1294
A	**671.3511**	336.1792	**482.2245**	241.6159
G	**728.3726**	364.6899	411.1874	206.0974
A	**799.4097**	**400.2085**	354.1660	177.5866
T	**900.4574**	**450.7323**	**283.1288**	142.0681
Y	1063.5207	**532.2640**	**182.0812**	91.5442

Supplementary Figure S9: MSMS-spectrum including the pMRM-transitions and sequence coverage (table; detected fragment ions are highlighted in bold) for the marker peptide P-ME16 (IKWGDAGATY; m/z 541.270; 2-fold charged; UniProt: G3P_PIG) in pork after thermolysin digest.

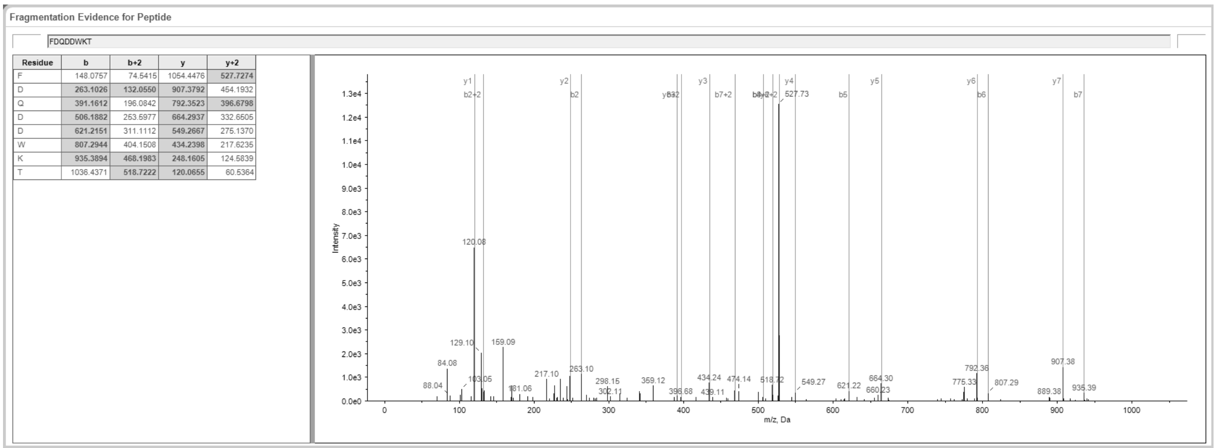

Residue	b	b+2	y	y+2
F	148.0757	74.5415	1054.4476	**527.7274**
D	**263.1026**	**132.0550**	**907.3792**	454.1932
Q	**391.1612**	196.0842	**792.3523**	**396.6798**
D	**506.1882**	253.5977	**664.2937**	332.6505
D	**621.2151**	311.1112	**549.2667**	275.1370
W	**807.2944**	404.1508	**434.2398**	217.6235
K	**935.3894**	**468.1983**	**248.1605**	124.5839
T	1036.4371	**518.7222**	**120.0655**	60.5364

Supplementary Figure S10: MSMS-spectrum including the pMRM-transitions and sequence coverage (table; detected fragment ions are highlighted in bold) for the marker peptide P-ME17 (FDQDDWKT; m/z 527.727; 2-fold charged; UniProt: ENOB_PIG) in pork after thermolysin digest.

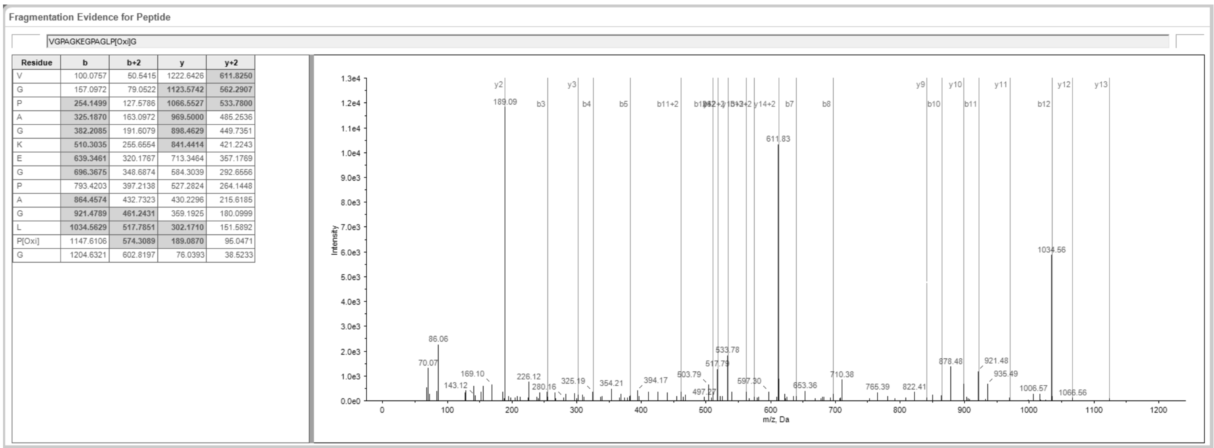

Residue	b	b+2	y	y+2
V	100.0757	50.5415	1222.6426	**611.8250**
G	157.0972	79.0522	**1123.5742**	**562.2907**
P	**254.1499**	127.5786	**1066.5527**	**533.7800**
A	**325.1870**	163.0972	**969.5000**	485.2536
G	**382.2085**	191.6079	**898.4629**	449.7351
K	**510.3035**	255.6554	**841.4414**	421.2243
E	**639.3461**	320.1767	713.3464	357.1769
G	**696.3675**	348.6874	584.3039	292.6556
P	793.4203	397.2138	527.2824	264.1448
A	**864.4574**	432.7323	430.2296	215.6185
G	**921.4789**	**461.2431**	359.1925	180.0999
L	**1034.5629**	**517.7851**	**302.1710**	151.5892
P[Oxi]	1147.6106	**574.3089**	**189.0870**	95.0471
G	1204.6321	602.8197	76.0393	38.5233

Supplementary Figure S11: MSMS-spectrum including the pMRM-transitions and sequence coverage (table; detected fragment ions are highlighted in bold) for the marker peptide P-SK09 (VGPAGKEGPAGLP[Oxi]G; m/z 611.825; 2-fold charged; UniProt: A0A4X1U043_PIG) in pig skin after thermolysin digest.

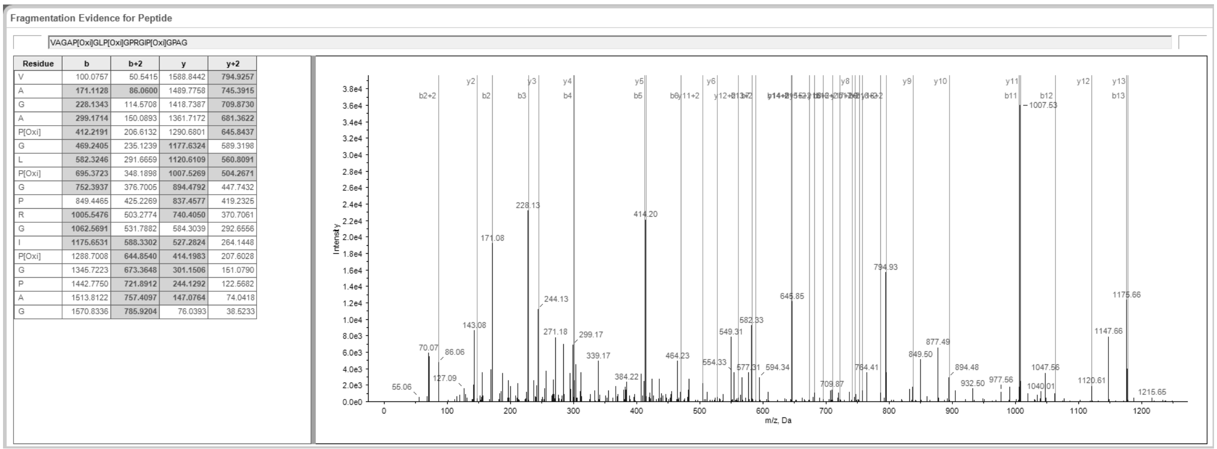

Supplementary Figure S12: MSMS-spectrum including the pMRM-transitions and sequence coverage (table; detected fragment ions are highlighted in bold) for the marker peptide P-SK10 (VAGAP[Oxi]GLP[Oxi]GPRGIP[Oxi]GPAG; m/z 794.926; 2-fold charged; UniProt: A0A4X1U043_PIG) in pig skin after thermolysin digest.

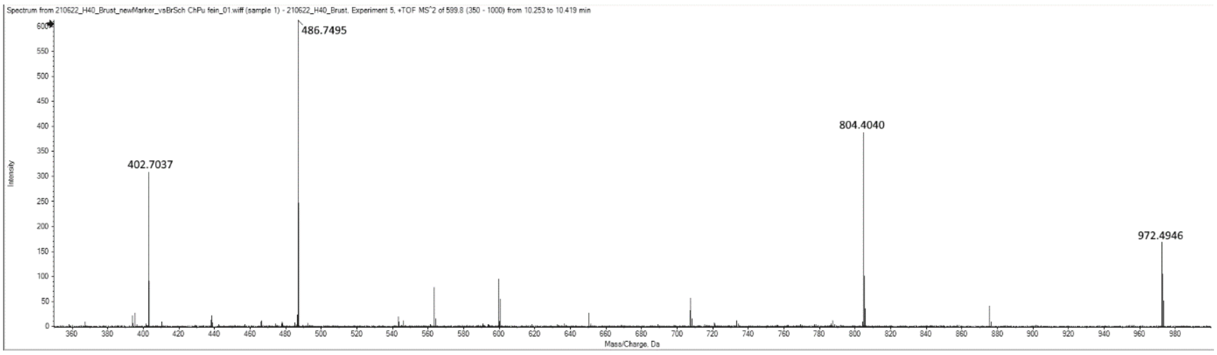

Supplementary Figure S13: MSMS-spectrum for the marker peptide C-ME06 (LLPAPGSYPYGRA; m/z 599.822; 2-fold charged; UniProt: HSPB1_CHICK) in chicken breast meat after thermolysin digest with optimized CE (28 V) showing the pMRM-transitions for identification in unknown samples.

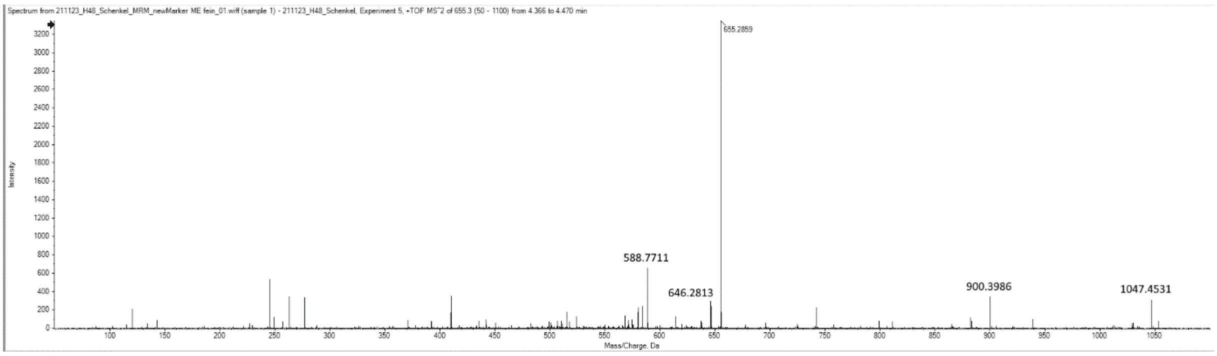

Supplementary Figure S14: MSMS-spectrum for the marker peptide C-ME09 (no sequence; m/z 655.285; 2-fold charged) in chicken breast meat after thermolysin digest with optimized CE (27 V) showing the pMRM-transitions for identification in unknown samples.

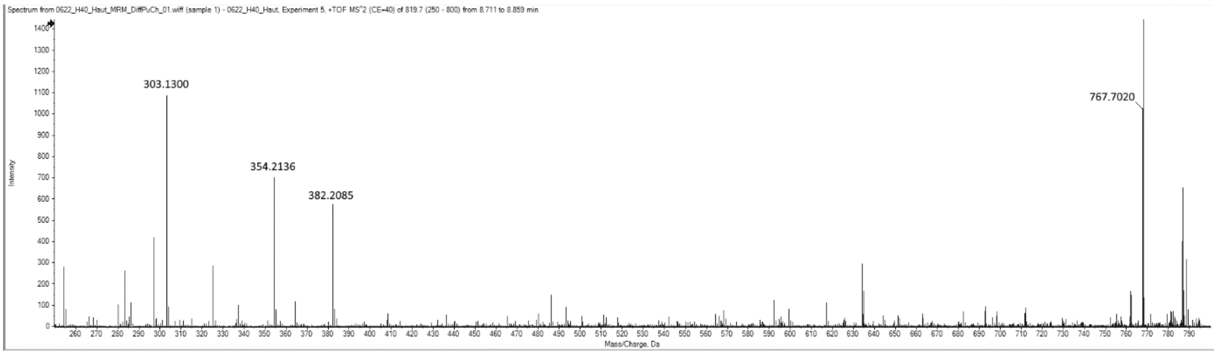

Supplementary Figure S15: MS/MS-spectrum for the marker peptide C-SK04 (VGPAGPIGSRGPSGPP[Oxi]GPDGNKGEP[Oxi]GN; m/z 819.730; 3-fold charged; UniProt: CO1A2_CHICK) in chicken skin after thermolysin digest with optimized CE (40 V) showing the pMRM-transitions for identification in unknown samples.

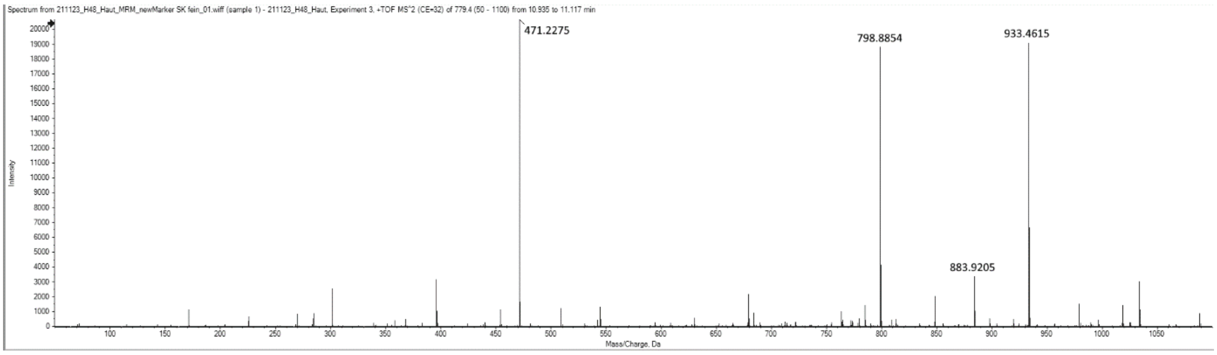

Supplementary Figure S16: MS/MS-spectrum for the marker peptide C-SK11 (VAVPGPMGPAGPRGLP[Oxi]GPP[Oxi]GAP[Oxi]GPQG; m/z 779.399; 3-fold charged; UniProt: CO1A1_CHICK) in chicken skin after thermolysin digest with optimized CE (32 V) showing the pMRM-transitions for identification in unknown samples.

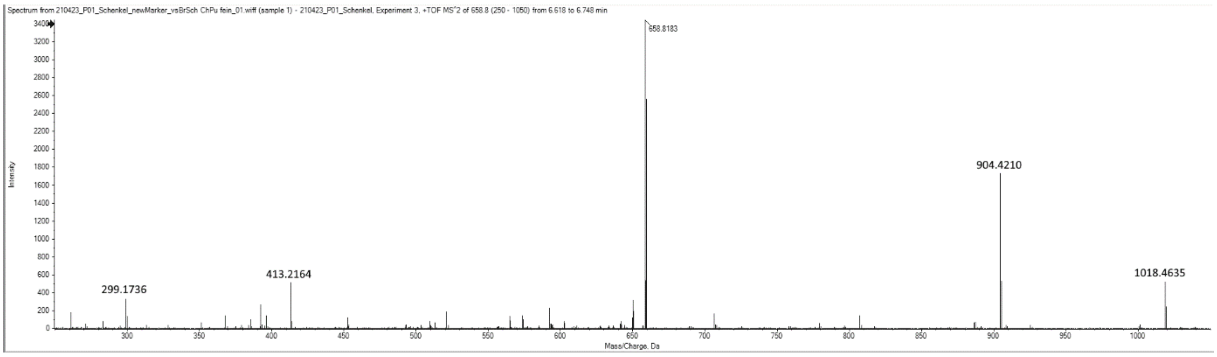

Supplementary Figure S17: MS/MS-spectrum for the marker peptide T-ME05 (LGQNPTNAEMNK; m/z 658.817; 2-fold charged; UniProt: A0A8B6WYV6_MELGA) in turkey thigh after thermolysin digest with optimized CE (30 V) showing the pMRM-transitions for identification in unknown samples.

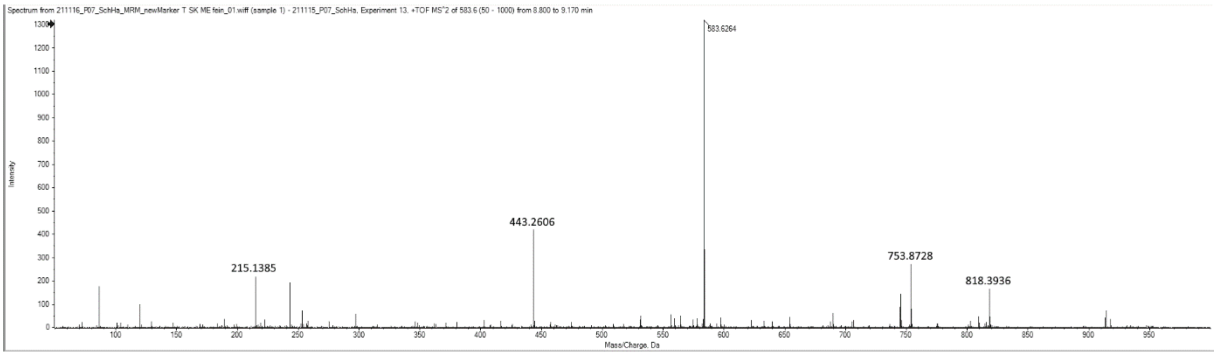

Supplementary Figure S18: MS/MS-spectrum for the marker peptide T-ME12 (no sequence; m/z 583.628; 3-fold charged) in turkey thigh after thermolysin digest with optimized CE (22 V) showing the pMRM-transitions for identification in unknown samples.

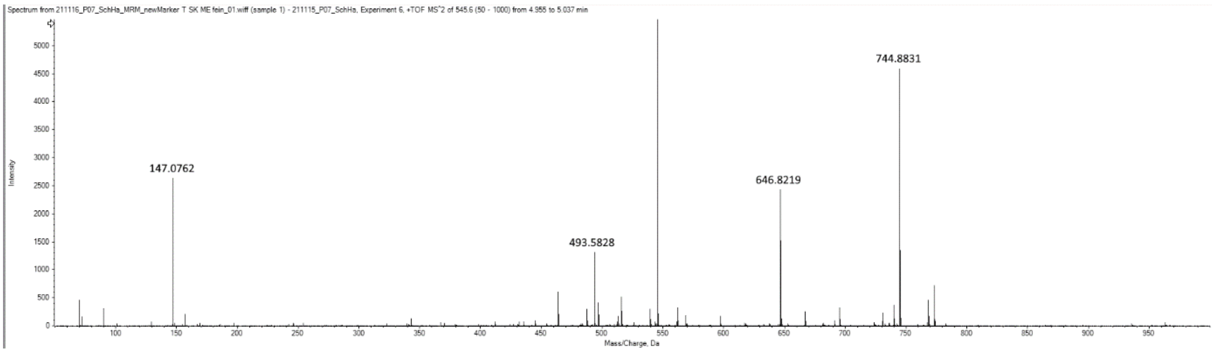

Supplementary Figure S19: MS/MS-spectrum for the marker peptide T-SK13 (no sequence; m/z 545.617; 3-fold charged) in turkey skin after thermolysin digest with optimized CE (20 V) showing the pMRM-transitions for identification in unknown samples.

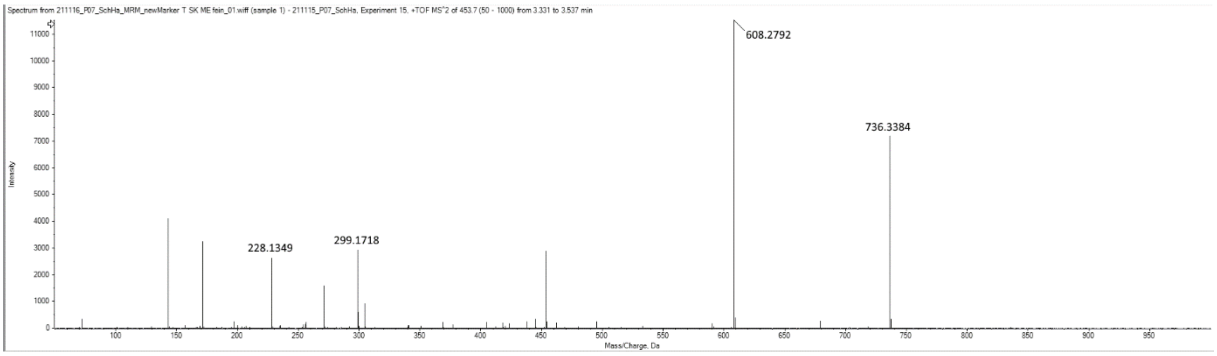

Supplementary Figure S20: MS/MS-spectrum for the marker peptide T-SK14 (no sequence; m/z 453.730; 2-fold charged) in turkey skin after thermolysin digest with optimized CE (20 V) showing the pMRM-transitions for identification in unknown samples.

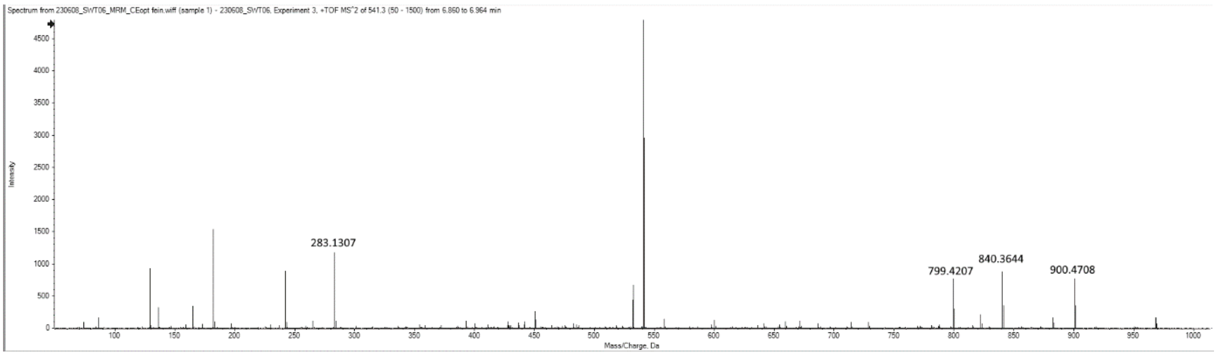

Supplementary Figure S21: MS/MS-spectrum for the marker peptide P-ME16 (IKWGDAGATY; m/z 541.270; 2-fold charged; UniProt: G3P_PIG) in pork after thermolysin digest with optimized CE (23 V) showing the pMRM-transitions for identification in unknown samples.

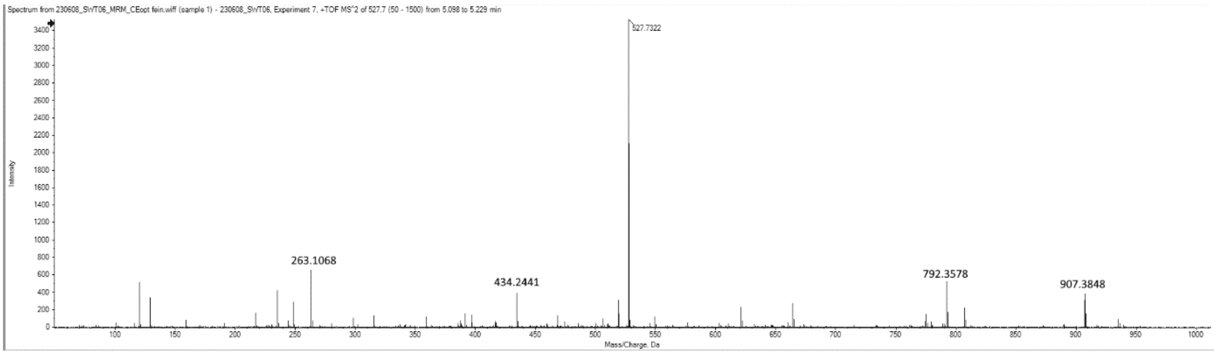

Supplementary Figure S22: MS/MS-spectrum for the marker peptide P-ME17 (FDQDDWKT; m/z 527.727; 2-fold charged; UniProt: ENOB_PIG) in pork after thermolysin digest with optimized CE (22 V) showing the pMRM-transitions for identification in pork after thermolysin digest in unknown samples.

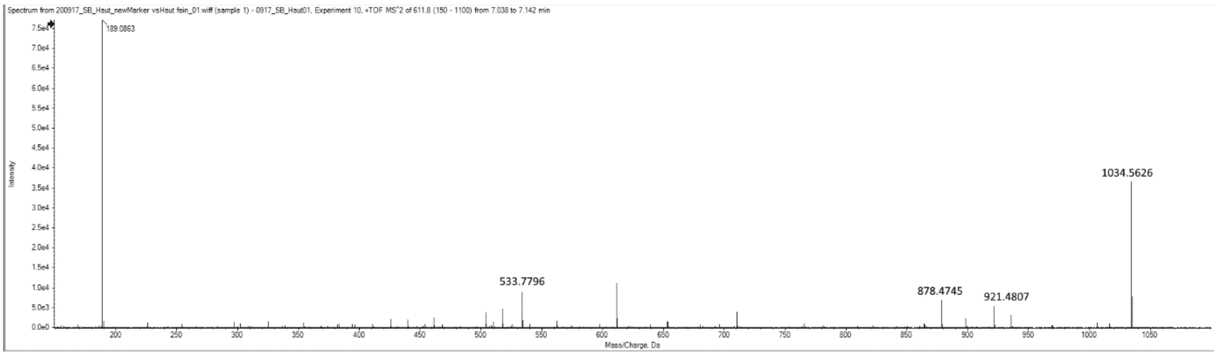

Supplementary Figure S23: MS/MS-spectrum for the marker peptide P-SK09 (VGPAGKEGPAGLP[Oxi]G; m/z 611.825; 2-fold charged; UniProt: A0A4X1U043_PIG) in pork skin after thermolysin digest with optimized CE (30 V) showing the pMRM-transitions for identification in unknown samples.

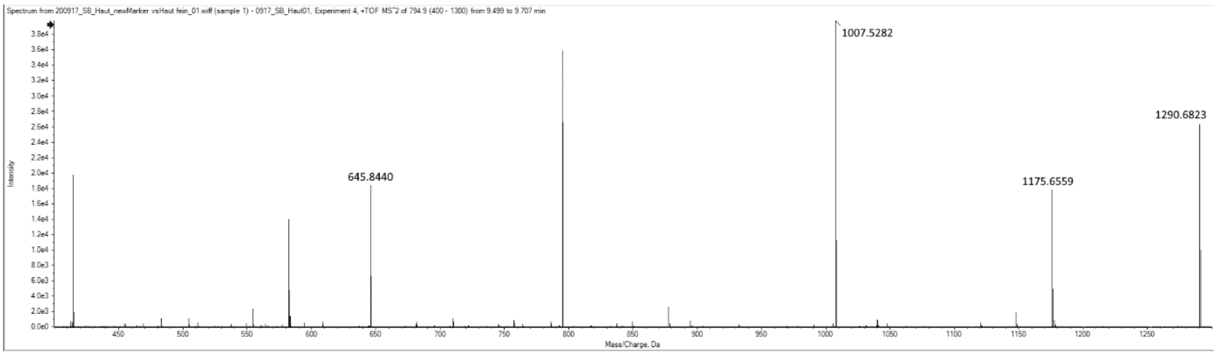

Supplementary Figure S24: MS/MS-spectrum for the marker peptide P-SK10 (VAGAP[Oxi]GLP[Oxi]GPRGIP[Oxi]GPAG; m/z 794.926; 2-fold charged; UniProt: A0A4X1U043_PIG) in pork skin after thermolysin digest with optimized CE (34 V) showing the pMRM-transitions for identification in unknown samples.
